# Strain Dependent Genetic Networks for Antibiotic-Sensitivity in a Bacterial Pathogen with a Large Pan-Genome

**DOI:** 10.1371/journal.ppat.1005869

**Published:** 2016-09-08

**Authors:** Tim van Opijnen, Sandra Dedrick, José Bento

**Affiliations:** 1 Boston College, Biology Department, Chestnut Hill, Massachusetts, United States of America; 2 Boston College, Computer Science Department, Massachusetts, United States of America; University of Tubingen, GERMANY

## Abstract

The interaction between an antibiotic and bacterium is not merely restricted to the drug and its direct target, rather antibiotic induced stress seems to resonate through the bacterium, creating selective pressures that drive the emergence of adaptive mutations not only in the direct target, but in genes involved in many different fundamental processes as well. Surprisingly, it has been shown that adaptive mutations do not necessarily have the same effect in all species, indicating that the genetic background influences how phenotypes are manifested. However, to what extent the genetic background affects the manner in which a bacterium experiences antibiotic stress, and how this stress is processed is unclear. Here we employ the genome-wide tool Tn-Seq to construct daptomycin-sensitivity profiles for two strains of the bacterial pathogen *Streptococcus pneumoniae*. Remarkably, over half of the genes that are important for dealing with antibiotic-induced stress in one strain are dispensable in another. By confirming over 100 genotype-phenotype relationships, probing potassium-loss, employing genetic interaction mapping as well as temporal gene-expression experiments we reveal genome-wide conditionally important/essential genes, we discover roles for genes with unknown function, and uncover parts of the antibiotic’s mode-of-action. Moreover, by mapping the underlying genomic network for two query genes we encounter little conservation in network connectivity between strains as well as profound differences in regulatory relationships. Our approach uniquely enables genome-wide fitness comparisons across strains, facilitating the discovery that antibiotic responses are complex events that can vary widely between strains, which suggests that in some cases the emergence of resistance could be strain specific and at least for species with a large pan-genome less predictable.

## Author Summary

While antibiotic resistant bacterial pathogens cause millions of deaths each year it remains largely unclear how a bacterium deals with antibiotic-induced stress and how this leads to the emergence of resistance. Moreover, many bacterial species are composed of strains whose genomes vary considerably, and while this variation may significantly affect phenotypes such as antibiotic sensitivity, its importance is unknown. Here we apply the method Tn-Seq, showing it is feasible to develop a detailed view of how a bacterium experiences antibiotic stress, while simultaneously determining the influence of the genomic-background. We show for two strains of the bacterial pathogen *Streptococcus pneumoniae* that, even though they experience the same stress triggered by daptomycin, they use a majority of different genes to withstand this stress, including genes important for integrity of the membrane, potassium uptake and protein turnover. Additionally, by untangling underlying genomic networks we unexpectedly expose large differences in genetic-interactions as well as transcriptional regulation. Our study provides not only a sensitive approach to untangle the influence of the genomic-background on phenotypes such as antibiotic sensitivity, but also highlights that this knowledge is instrumental in understanding how bacteria respond to environmental stress, which in turn influences the manner in which they evolve.

## Introduction

Bacteria evolve antibiotic resistance in response to selective pressures that emerge from the interaction between the antibiotic and the bacterium. Routes of escape often lead through ways that diminish the interaction with the direct target. For instance, escape from penicillin, whose direct target are the Penicillin Binding Proteins (PBPs), can often be found in mutations in PBPs that decrease the affinity for the drug, or in functionally related genes that compensate for diminished function [1–4]. However, it has become clear that the relationship between a bacterium and an antibiotic reaches far beyond its direct target. Instead an antibiotic triggers a complex, multi-factorial process that may begin with the physical interaction between the drug and its target but quickly propagates into the involvement of a variety of processes that can include regulation, metabolism and/or energy generation [5–12]. These system-wide selective pressures could explain why clinical strains often contain multiple alterations that may contribute to resistance but are located in genes whose primary role is not resistance but rather are involved in fundamental bacterial processes [13–17]. The adaptive sequence space thus seems to lie well beyond the antibiotic’s direct target, which contributes to the complexity of determining and predicting how and where resistance evolves. Moreover, adaptive mutations can be species-specific. For instance, the direct target of fluoroquinolones in gram-negatives including *Escherichia coli* is DNA gyrase, while in gram positives such as *Staphylococcus aureus* it is topoisomerase IV, which may explain why mutations in *gyrA* such as S83L in *E*. *coli* can increase the MIC to fluoroquinolones while the equivalent mutation in *gyrA* in *S*. *aureus* does not necessarily have an effect on the MIC [18–23]. Additionally, some *gyrA* mutations are associated with a fitness cost in *E*. *coli* [24, 25], while a positive fitness effect can be observed in *Campylobacter jejuni* and *Salmonella enterica* [26, 27]. These contrasting phenotypes suggest that the manner in which a bacterium experiences antibiotic-induced stress may differ depending on the genomic background and the underlying genomic network. While detailed insights into these factors could help in designing novel antimicrobial strategies, the importance of the genomic background and to which extent antibiotic-sensitivity and resistance depend on network architecture is currently unclear.

In this study we use *Streptococcus pneumoniae* to explore the importance of the genomic background on antibiotic-sensitivity and the manner in which stress is experienced and processed. *S*. *pneumoniae* is a human nasopharyngeal commensal and respiratory pathogen. It triggers pneumococcal pneumonia, meningitis, and septicemia, which results in ~1 million deaths annually among children <5 years of age, and ~0.5 million among groups including the immunocompromised and the elderly (>65 yrs.), making it one of the most important bacterial pathogens worldwide [28–30]. Although vaccination has been successful, we and others have shown that it does not result in complete protection, and that some groups, such as children with Sickle Cell Disease, remain especially vulnerable [31]. Antibiotics thus continue to be extremely important as a treatment option, especially in acute disease. However, as with almost any clinically important bacterial pathogen, the emergence of multidrug-resistant (MDR) strains is a global problem [32–37] and with 1.2 million drug-resistant pneumococcal infections annually in the US, and $96 million in excess medical costs, *S*. *pneumoniae* is a serious concern [30]. *S*. *pneumoniae* is one of several species for which the availability of complete bacterial genomes has demonstrated that a distinction can be made between its core-genome (the pool of genes shared by all members of a species) and pan-genome (a species’ global gene repertoire) [38–41]. On average two pneumococcal strains may differ by ~300 genes in their genomic content, *i*.*e*. the presence and absence of genes [42, 43], which highlights the genome’s plasticity to retain function in the presence of variation. Such plasticity is remarkable because no genomic element, gene, or pathway exists in a vacuum; rather they are connected through networks resulting in specific organismal properties [44, 45]. A newly acquired element thus needs to be integrated thereby possibly affecting existing connections and creating new ones. Consequently, no two genomes may function in the same manner, potentially affecting phenotypes ranging from drug tolerance to virulence to evolutionary potential.

By employing genome-wide approaches we, and others, have shown that it is possible to determine, upon exposure to an environmental perturbation, where stress in the bacterial genome is experienced [31, 46–55]. Here we apply Tn-Seq, a tool for systems-level analysis of microorganisms, which combines transposon mutagenesis with massively parallel sequencing to determine genome-wide fitness in a single experiment. We develop daptomycin-sensitivity profiles for two strains of the bacterial pathogen *S*. *pneumoniae*. Although the exact mechanism of action of daptomycin is not completely clear it seems to insert itself into the membrane for which the presence of phosphatidylglycerol in the membrane is required. Following insertion, membrane structure and curvature may be distorted leading to cells with altered cell shapes. These distortions in the membrane at the site of daptomycin insertion may lead to leakage of ions and loss of membrane potential and local dysregulation of cell division and/or cell wall-biosynthesis [56–61]. The daptomycin-sensitivity profiles generated in this study illustrate how the antibiotic’s effects ripple through the organism and how the bacterium deals with this stress with a diverse set of genes from different functional categories and organizational levels including: cell-wall organization, membrane integrity and transport, control and regulation of fundamental processes, and metabolism. Surprisingly, the sensitivity profiles turn out to be highly strain-specific highlighted by over 50% of sensitivity-profile genes that increase antibiotic sensitivity in one strain but have no effect, or even decrease sensitivity in the other strain. We show that these differences are partially the result of a network architecture that is not well conserved, exemplified by strain-specific differences in Potassium (K^+^)-release and ClpP functionality, as well as differences in regulatory relationships between genes from different organizational levels. Importantly, we present a generally applicable, and highly sensitive approach that enables comparisons of environment-induced fitness effects on a genome-wide scale and species-wide level.

## Results and Discussion

### Two *S*. *pneumoniae* strains with variable gene content have the same susceptibility to daptomycin

**Fig 1:**
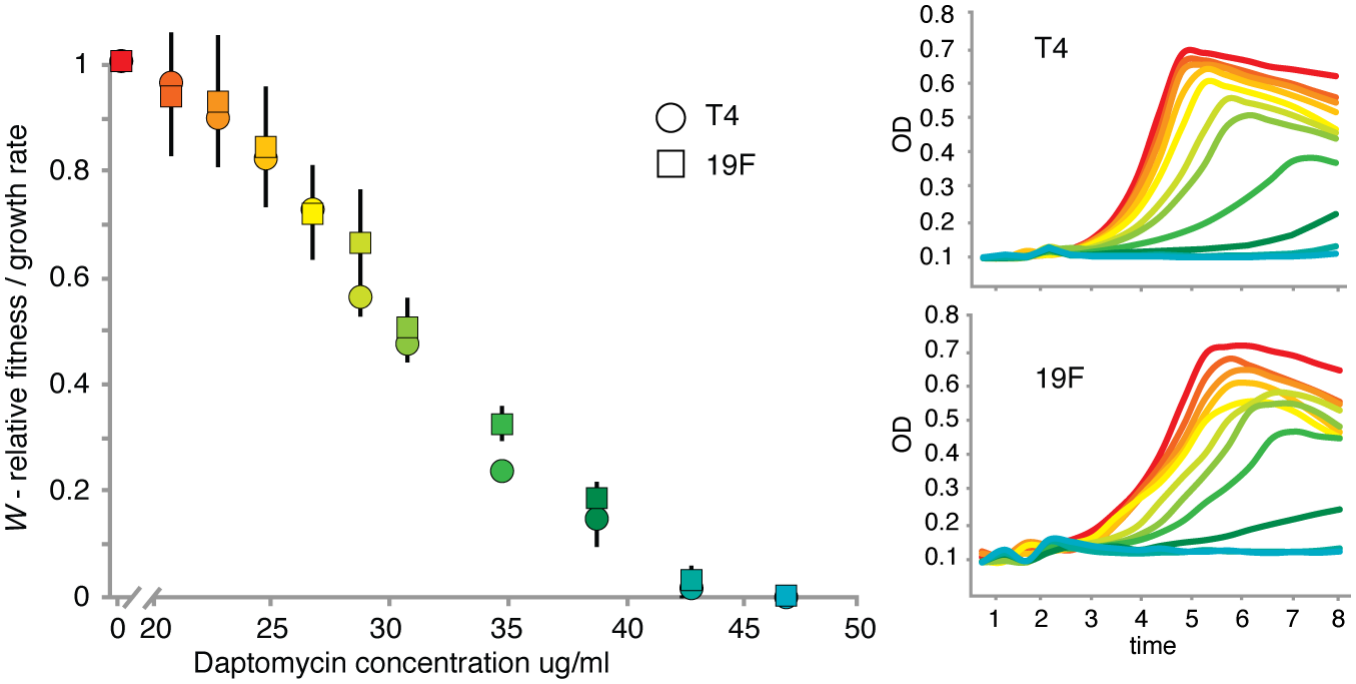
Daptomycin growth rate reduction in T4 and 19F. The left panel shows that the reduction in the growth rate with increasing daptomycin concentration is the same for strains T4 (circles) and 19F (squares). For instance a concentration of 25 μg/ml daptomycin results in a relative fitness (*W* ± standard deviation) of 0.83 ± 0.04 for T4 and 0.85 ± 0.11 for 19F, which means that the growth rate under these circumstances increases on average by 17% and 15% respectively. For illustrative purposes, a fitness of 0.5 indicates that the relative growth rate increases by 50% and that the doubling time is 2-fold longer compared to a ‘standard’ or in this case the no-antibiotic condition. The right two panels depict representative growth curves for each strain in the absence and presence of increasing daptomycin concentrations. Colors refer to the used daptomycin concentration and are the same for all three figures, with red being no daptomycin (*W* = 1.0), and turquoise depicting the highest concentration of daptomycin at 47ug/ml (*W* = 0).

A major goal of this study is to determine whether bacterial strains from the same species that differ in their genomic content, respond in an identical manner to antibiotic stress. On a species level the genome of *S*. *pneumoniae* can be divided up in a core genome consisting of ~1600 genes, and a pan genome of ~4000 genes, while a genome on average has approximately 2000 genes. We selected two strains, TIGR4 (T4) and Taiwan-19F (19F) (S1 Fig), which can both cause invasive disease: T4 is a serotype 4 strain that was originally isolated from a patient from Norway with Invasive Pneumococcal Disease (IPD) [62, 63], while 19F is a multi-drug resistant (MDR) strain isolated from a patient with IPD from Taiwan [64, 65]. With respect to genomic content the strains share 1711 genes, while T4 has 324 genes that are absent in 19F, and 19F has 204 genes that are absent in T4. On average two pneumococcal strains may differ by ~15% in their genomic content, and thus the amount of variation between these two strains is representative for differences observed between strains within the species [42, 43]. All genes were split into 17 functional categories and except for the number of genes with unknown function, both strains share a similar distribution over these categories (S2 Fig; S1 Table). Both strains are differentially susceptible to different antibiotics, for instance 19F is approximately 25-fold less susceptible to penicillin than T4, while they are equally sensitive to daptomycin (Fig 1). Because equal sensitivity creates the simplest opportunity to test whether strains use the same genes in dealing with antibiotic induced stress, daptomycin is used here.

### Antibiotic sensitivity profiles reveal antibiotic functionality and leads to gene function

**Fig 2:**
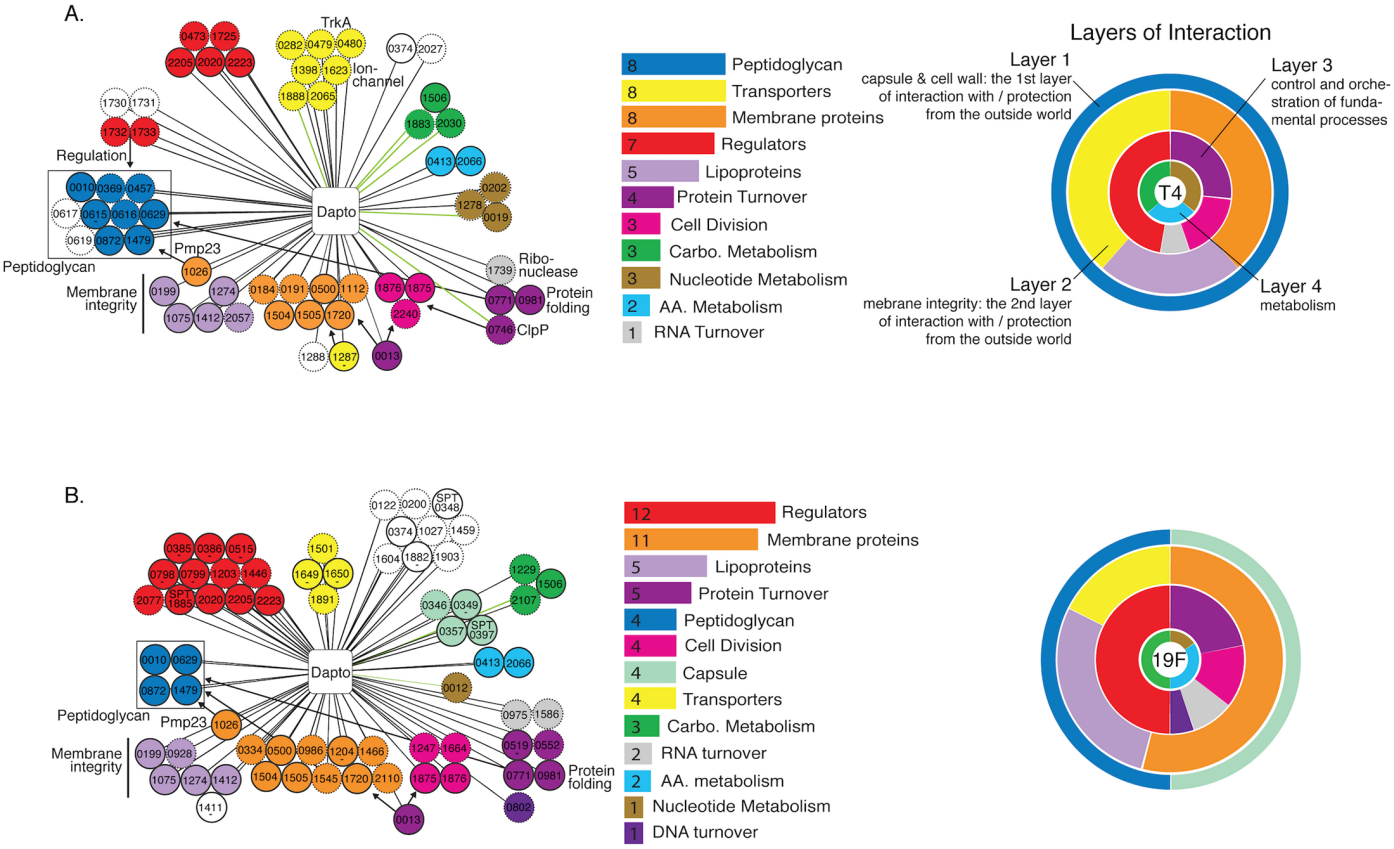
Strain-specific daptomycin-sensitivity profiles for T4 (A) and 19F (B). Each color-coded circle (node) represents a gene depicted by its gene number (note that all numbering is according to strain T4; for strain specific numbers see the correspondence table in S8 Table), that is connected by a line (edge) to daptomycin in the center of the network. The edge indicates that the gene’s fitness significantly changes in the presence of daptomycin; a black edge indicates fitness is significantly lower, while a green edge indicates fitness increases. A node with a dotted border indicates that its phenotype is not conserved in the other strain, while a solid black border indicates its phenotype is conserved, unless there is a dash underneath the gene-number which means it was impossible to determine whether the phenotype was conserved in the other strain due to too little insertions. If a gene-number starts with SP or SPT, the gene is strain-specific in T4 or 19F respectively. Some sets of genes are highlighted in the networks by indicating their functional role (*e*.*g*. peptidoglycan, ClpP) or by arrows that represent relationships (e.g. SP1732/SP1733 have a regulatory effect on peptidoglycan). Genes with unknown function are depicted in white, and if they have a close neighbor with an annotated function in the network they are placed next to that neighbor, possibly suggesting a shared role. Next to each network the importance of each functional category in the network is ranked by the number of genes present in the network. Finally, as described in more detail in the main text, and to illustrate that the antibiotic interacts with the bacterium on different organizational levels, each functional category belongs to one of four hierarchical layers which combines the physical location of the gene-product with its molecular function. The first layer consists of capsule and peptidoglycan genes and is both the first layer of protection from, and interaction with, the outside world. The second layer is important for membrane integrity and transport across the membrane represented by membrane, lipoprotein and transporter genes. The third layer controls and orchestrates fundamental processes including cell division, transcription and translation and regulation, while the fourth combines all metabolism genes. The importance of each layer is highlighted by the thickness of the layer, which is determined by the number of genes in that layer, while within each layer the importance of each category is represented as a percentage.

To identify in detail which genes in the genome are involved in dealing with daptomycin-stress we employed transposon insertion sequencing (Tn-Seq), which enables high-throughput and accurate calculations of the growth rate for each possible gene-knockout in the genome [31, 46, 48, 49]. Six independent transposon libraries, each consisting of ~10,000 mutants were created in T4 and were grown in the absence and presence of daptomycin at a concentration of 25 μg/ml, which moderately slows the growth rate by ~15% (Fig 1). For each condition reproducibility was determined by comparing fitness between different libraries, which in each case was high (R^2^ = 0.78–0.89). Fitness values for each insertion in each gene were averaged and genes with a significant antibiotic-specific response were visualized in a network with Cytoscape [66] and grouped according to their functional category (Fig 2A; S2 Table). Previously we showed how such a visual network approach provides a detailed overview of how an environmental disturbance can affect a bacterium on multiple different levels [31, 48]. The same is true for this network, demonstrating how a large variety of genes from different functional categories become important for the survival of T4 in the presence of daptomycin (Fig 2A; S2 Table), which highlights several aspects of daptomycin’s *modus operandi*: 1) Our results show that any gene that affects peptidoglycan (PG) biosynthesis, stability, or regulation can, upon its removal, make the bacterium more susceptible to daptomycin. Even a decrease in PG acetylation (mediated by SP1479) or a decrease in the speed and efficiency of Penicillin Binding Protein (PBP) folding, which has been shown to be mediated by PrsA in *B*. *subtilis* [67] (*prsA*/SP0981), increases susceptibility to daptomycin. Associations of daptomycin with the cell wall have also been shown in other bacteria: in *B*. *subtilis*, and *S*. *aureus*, daptomycin induces a cell wall stress response [59, 68], and mutations that increase cell wall thickening have been associated with resistance in *S*. *aureus*. [69, 70]; 2) Lipo- and membrane proteins that provide structural support to the membrane become important in the presence of daptomycin, suggesting that the interaction of daptomycin with the membrane has a de-stabilizing effect on membrane integrity. The importance of the membrane anchored protease FtsH (SP0013), whose function includes proteolysis of aberrant membrane proteins and thereby influences membrane turnover [71, 72], further suggests that daptomycin negatively affects membrane-protein stability and thus membrane integrity. In *B*. *subtilis*, daptomycin preferentially interacts with regions of the membrane enriched in phosphatidylglycerol (PhG) [59], it has been physically associated with sites of membrane distortion [56] and resistance is linked to the overall PhG content [73], in *S*. *aureus* mutations in *mprF* increase daptomycin resistance by changing membrane lipid composition and charge [74–76], while in Enterococci changes in cardiolipin synthesis can increase daptomycin resistance [77, 78]; 3) A Trk-system (SP0479-0480), which mediates K^+^-uptake [79], becomes important in T4 in the presence of daptomycin, which suggests that T4 suffers from daptomycin-induced K^+^-loss. Indeed, it is assumed that daptomycin triggers potassium loss through its interaction with the membrane [57, 60, 61]. Besides K^+^, other ions may be leaking out as well [61], or at least other ions become more important and may compensate for K^+^-loss, which is highlighted by the importance of several ion transport systems in our network including SP1623 (annotated as a cation-transporter), which we previously associated with pH-homeostasis [48]; 4) The sensitivity profile highlights the importance of a diverse set of cell division, RNA and protein turnover, signaling and regulation, and metabolism genes, indicating that the antibiotic’s effects resonate throughout some of the most embedded systems in the bacterium. Importantly, these profiles can also suggest roles for genes with unknown or unclear functions in at least two ways: 1) A gene with unknown function adjacent to a gene with a defined function and a similar sensitivity suggests that the two genes are involved in the same process. For instance SP1730 and SP1731 are hypothetical genes and have a similar fitness as their regulatory neighbors SP1732 (*stkP*) and SP1733 (*phpP*; the cognate phosphatase of *stkP*), which sense intracellular peptidoglycan and have regulatory control over cell-division [80]. 2) Genes with domains that suggest a function or association with a specific functional category are more likely to be correct if they fit within the sensitivity profile. For instance BLAST searches and protein domain predictions predict that both SP1505 and SP1720 are membrane proteins. These predictions fit well with the sensitivity profile where membrane proteins make up one of the most important categories. Moreover, with GFP-fusions we confirmed the localization of both proteins in the membrane.

### Daptomycin sensitivity profiles are not conserved between strains

**Fig 3:**
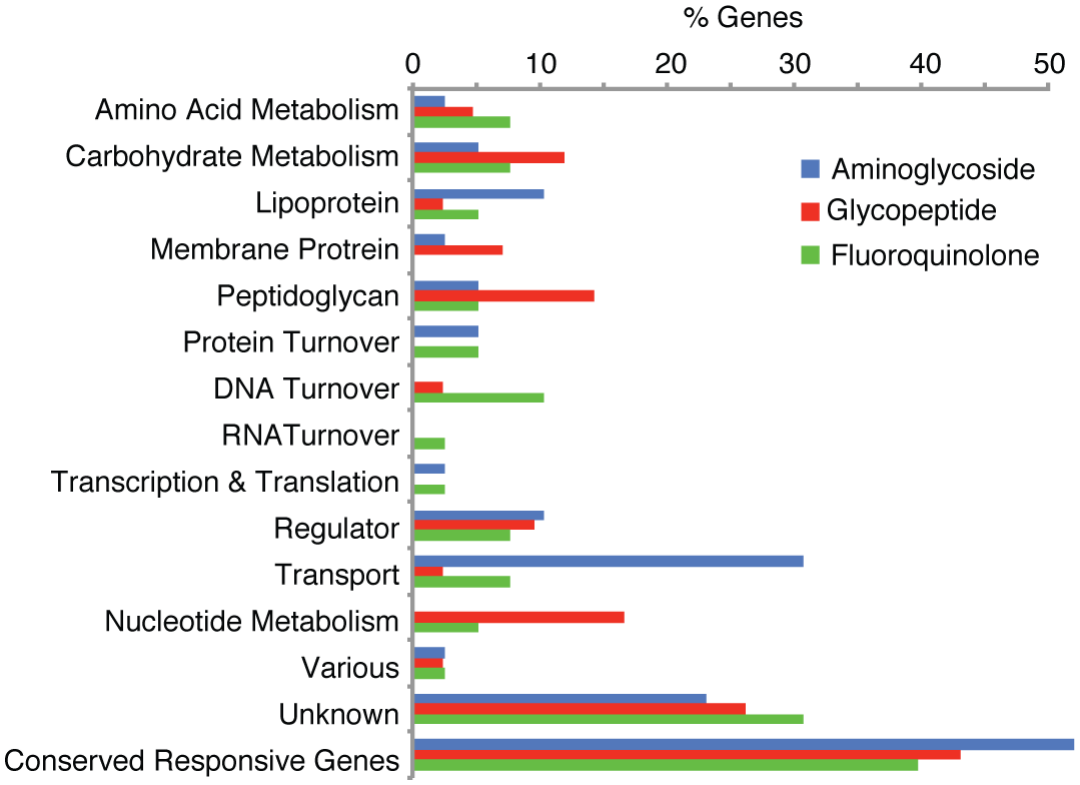
Tn-Seq on T4 and 19F in the presence of three different classes of antibiotics. A Tn-Seq generated distribution in functional categories of genes that are important in the presence of three different classes of antibiotics shows that, similar to daptomycin, these classes of antibiotics also trigger stress in the bacterium that is dealt with by genes from a large variety of functional categories. Moreover, the category “Conserved Responsive Genes” depicts the percentage of genes that are used in both T4 and 19F to deal with the stress triggered by the respective antibiotic, indicating that similar to daptomycin, conservation is limited and ranges between 40–50%.

We expected to uncover highly similar sensitivity profiles due to the strains’ equivalent susceptibility to daptomycin. However, less than 50% of the responsive genes have a conserved phenotype between strains (Fig 2B; S3 Table), and based on a Jaccard similarity index, the networks are significantly different (*J* = 0.24, p<0.05)[81–83]. Additionally, the overall distribution of functional gene categories is significantly different between strains (*two proportion exact test*; Z = 5.83, N_1_ = 52 N_2_ = 57, p<0.01). This means that both on the individual gene-level as well as the overall functional level there is little conservation between strains in the distribution of the type of genes that are important in dealing with daptomycin stress. However, genes and pathways interact with each other and responses could be more conserved on a global scale. Therefore we grouped functional categories to determine whether we could analytically track how an antibiotic interacts with a bacterium and thereby identify how a bacterium in first instance perceives a (extracellular) threat and subsequently how this threat is processed. To enable this, functional categories were combined into four hierarchical groups, or layers. The first layer combines categories that make up the first physical layer an antibiotic could interact with, which is the capsule and the cell wall represented by peptidoglycan genes. The second physical layer of interaction is represented by the membrane and consists of the categories membrane, lipoprotein and transporter genes. The third layer combines genes that control and orchestrate fundamental processes: cell division, DNA turnover, RNA turnover, protein turnover, transcription and translation and regulation. The fourth and last layer combines all metabolism genes including nucleotide, carbohydrate and amino acid metabolism. These four layers thus combine the physical location of the gene-product with its molecular function (Fig 2). For both strains the first layer is indeed the first point of interaction (Fig 2A and 2B) after which the membrane becomes the next obstacle. This second layer includes 21 responsive genes in T4 and 20 in 19F and even though only 25% of the transporter genes in this layer are conserved, ~70% of the membrane and lipoprotein genes are conserved between strains. Thus, at least for the part of the network that is important for membrane integrity the two strains seem to experience and process daptomycin stress in a similar fashion. Moreover, when a global analysis is performed, in which the gene-categories are first collapsed into the described four layers, and then the four layers are compared between the two strains, we no longer observe a dissimilar response (*two proportion exact test*, Z = 2.85, N_1_ = 52 N_2_ = 57, p = 0.16), indicating that although on the individual gene level there is little conservation, the global response is more similar. To confirm that the wide variety of genes involved in dealing with antibiotic stress, as well as the lack of phenotypic conservation is not limited to daptomycin we further performed Tn-Seq with an aminoglycoside, a glycopeptide and a fluoroquinolone, and in both T4 and 19F, which shows that also these three classes of antibiotics trigger stress that is processed with genes from a wide variety of categories (Fig 3). In addition, conservation of phenotypes, *i*.*e*. the genes that either strain uses to deal with antibiotic stress, shows, similar to daptomycin, a limited signature of conservation between ~40–50% (Fig 3).

### High confidence fitness results confirm lack of conservation in sensitivity profiles

**Fig 4:**
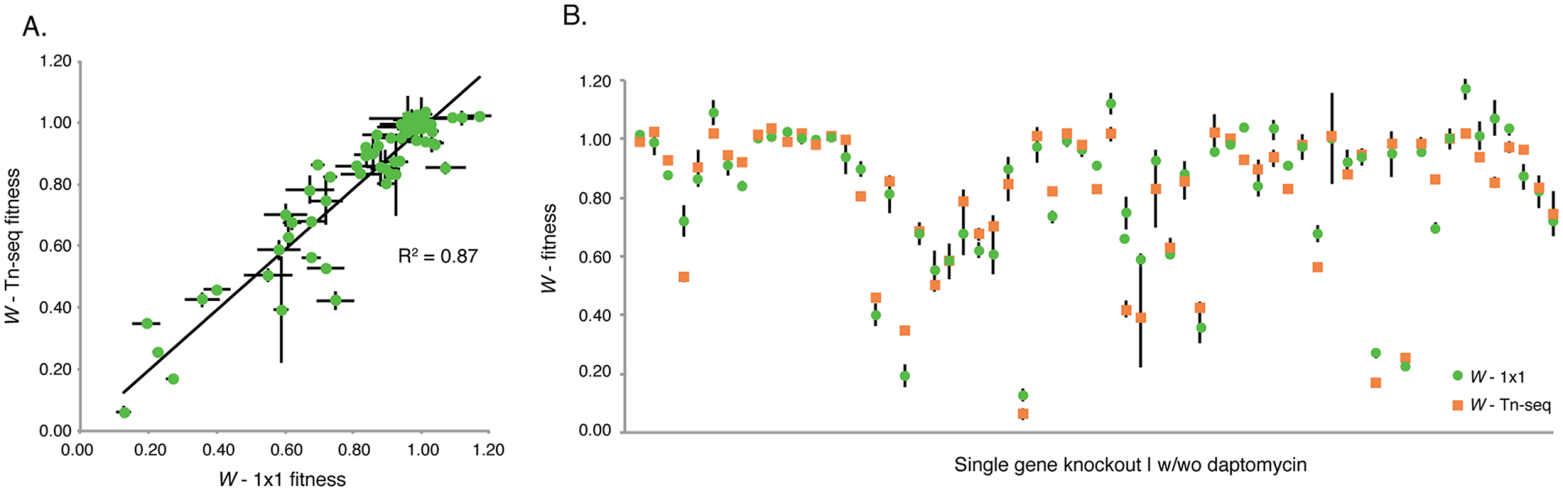
Sensitivity profile data of single gene knockouts correlates strongly with 1x1 competitions and growth curves. (A) The strong correlation between Tn-Seq fitness (*W*_Tn-Seq_) and fitness calculated from individual mutant growth curves or 1x1 competitions from both T4 and 19F mutants (*W*_1x1_; n = 65, shown are average ± SEM) emphasizes that the strain-dependent sensitivity profiles are composed of high-confidence Tn-Seq data. (B) No significant differences were found between Tn-Seq fitness and 34 deletion mutants that were individually verified in the presence or absence of daptomycin (65 genotype phenotype relationships; Table 1).

To validate the sensitivity profiles, and exclude that the lack of conservation comes from low confidence Tn-Seq data, we compared Tn-Seq fitness (*W*_Tn-Seq_) to fitness obtained from individual growth curves and/or from 1x1 competition assays (*W*_1x1_), in which a deletion-mutant is competed against the wild type. Note that in all three of these cases fitness (*W*) is calculated as the growth rate thereby enabling direct comparisons. In total 34 deletion mutations in T4 and 19F were constructed and sixty-five genotype-phenotype relationships were validated in the presence and absence of daptomycin (Fig 4A and 4B; Table 1). This resulted in a strong correlation (R^2^ = 0.87), which is similar to correlations we achieved previously [48] and confirms high-confidence Tn-Seq fitness data. Therefore, even though the strains have the same susceptibility to daptomycin, belong to the same species and share ~85% of their genomic content, this suggests that the underlying genomic networks must be different, which makes the strains respond in a different manner to the same stress.

**Table 1:** Fitness validation of single deletion mutants in T4 and 19F, with and without daptomycin.

Gene^a^	*W*- 1x1± s.e.^b^	*W*—Tn-Seq ± s.e^c^	1x1 n^d^	Tn-Seq n^e^	Strain^f^	Condition	Annotation / Layer / Role
SPT1006 (unique)	1.01 ± 0.04	0.99 ± 0.01	4	92	19F	-	Potassium channel / 2 / Transport
SPT1006 (unique)	0.99 ± 0.01	1.03 ± 0.01	4	92	19F	Daptomycin	
SP0013	0.87 ± 0.05	0.92 ± 0.02	4	38	19F	-	Cell division protease FtsH / 3 / Protein turnover
SP0013	0.72 ± 0.01	0.53 ± 0.06	4	38	19F	Daptomycin	
SP0019	0.86 ± 0.04	0.90 ± 0.01	3	47	T4	-	Adenylosuccinate synthetase / 4/ Nucleotide metabolism
SP0019	1.09 ± 0.04	1.02 ± 0.01	3	50	T4	Daptomycin	
SP0019	0.91 ± 0.01	0.95 ± 0.01	3	196	19F	-	
SP0019	0.84 ± 0.01	0.92 ± 0.01	3	183	19F	Daptomycin	
SP0047	1.00 ± 0.01	1.01 ± 0.01	4	33	T4	-	Phosphoribosylaminoimidazole synthetase / 4 / Nucleotide metabolism
SP0047	1.01 ± 0.01	1.03 ± 0.01	4	33	T4	Daptomycin	
SP0047	n.d. ^g^	1.01 ± 0.01		99	19F	-	
SP0047	1.03 ± 0.02	0.99 ± 0.01	4	96	19F	Daptomycin	
SP0078	1.00 ± 0.01	1.02 ± 0.01	4	109	T4	-	Trk potassium uptake Trk2 / 2 / Transport
SP0078	1.00 ± 0.01	0.98 ± 0.01	4	109	T4	Daptomycin	
SP0078	1.00 ± 0.06	1.00 ± 0.01	4	208	19F	-	
SP0078	0.94 ± 0.03	0.99 ± 0.01	4	208	19F	Daptomycin	
SP0199	0.90 ± 0.04	0.80 ± 0.01	4	136	T4	-	Cardiolipin synthetase / 2 / Lipoprotein
SP0199	0.40 ± 0.07	0.46 ± 0.01	4	136	T4	Daptomycin	
SP0199	0.81 ± 0.04	0.86 ± 0.01	4	328	19F	-	
SP0199	0.19 ± 0.04	0.35 ± 0.01	4	328	19F	Daptomycin	
SP0202	0.68 ± 0.07	0.68 ± 0.02	4	61	T4	-	Anaerobic ribonucleoside triphosphate reductase / 4 / Nucleotide metabolism
SP0202	0.55 ± 0.06	0.50 ± 0.03	4	61	T4	Daptomycin	
SP0202	n.d. ^g^	0.62 ± 0.04		56	19F	-	
SP0202	0.58 ± 0.07	0.59 ± 0.04	4	56	19F	Daptomycin	
SP0205	0.67 ± 0.03	0.78 ± 0.02	4	26	T4	-	Anaerobic ribonucleoside-triphosphate reductase activating protein / 4 / Nucleotide metabolism
SP0205	0.62 ± 0.06	0.68 ± 0.04	4	26	T4	Daptomycin	
SP0205	n.d. ^g^	0.79 ± 0.04		30	19F	-	
SP0205	0.60 ± 0.04	0.70 ± 0.06	4	30	19F	Daptomycin	
SP0457	0.89 ± 0.02	0.85 ± 0.02	4	17	T4	-	UDP pyrophosphate phosphatase / 1 / Peptidoglycan
SP0457	0.13 ± 0.05	0.06 ± 0.03	4	17	T4	Daptomycin	
SP0479	0.97 ± 0.02	1.01 ± 0.01	8	76	T4	-	Trk potassium uptake Trk1 / 2 / Transport
SP0479	0.73 ± 0.02	0.82 ± 0.01	8	76	T4	Daptomycin	
SP0479	0.99 ± 0.03	1.02 ± 0.01	4	207	19F	-	
SP0479	0.97 ± 0.02	0.98 ± 0.01	4	207	19F	Daptomycin	
SP0746	0.91 ± 0.04	0.83 ± 0.02	6	10	T4	-	ClpP / 3 / Protein turnover
SP0746	1.12 ± 0.06	1.02 ± 0.03	4	10	T4	Daptomycin	
SP0746	0.75 ± 0.02	0.42 ± 0.17	8	5	19F	-	
SP0746	0.59 ± 0.01	0.39 ± 0.13	8	5	19F	Daptomycin	
SP0798	0.93 ± 0.02	0.83 ± 0.03	4	11	T4	-	DNA-binding response regulator CiaR / 3 / Regulator
SP0798	0.61 ± 0.04	0.63 ± 0.06	4	11	T4	Daptomycin	
SP0798	0.88 ± 0.05	0.86 ± 0.02	4	27	19F	-	
SP0798	0.36 ± 0.01	0.42 ± 0.07	4	27	19F	Daptomycin	
SP1398	0.96 ± 0.01	1.02 ± 0.01	3	83	19F	-	Phosphate ABC transporter permease / 2 / Transport
SP1398	0.98 ± 0.01	1.00 ± 0.01	3	70	19F	Daptomycin	
SP1466	1.04 ± 0.04	0.93 ± 0.03	3	10	T4	-	Hemolysin/ 2 / Membrane
SP1466	0.84 ± 0.04	0.90 ± 0.03	3	8	T4	Daptomycin	
SP1466	1.03 ± 0.01	0.93 ± 0.01	3	80	19F	-	
SP1466	0.91 ± 0.04	0.83 ± 0.02	3	63	19F	Daptomycin	
SP1504	0.97 ± 0.03	0.98 ± 0.01	4	21	T4	-	Hypothetical Protein
SP1504	0.68 ± 0.16	0.56 ± 0.07	4	21	T4	Daptomycin	
SP1504	1.00 ± 0.04	1.01 ± 0.01	4	130	19F	-	
SP1504	0.92 ± 0.03	0.87 ± 0.02	4	130	19F	Daptomycin	
SP1505	0.94 ± 0.02	0.95 ± 0.01	4	47	T4	-	Hypothetical Protein
SP1505	0.27 ± 0.08	0.17 ± 0.03	4	47	T4	Daptomycin	
SP1505	0.95 ± 0.02	0.98 ± 0.01	4	149	19F	-	
SP1505	0.23 ± 0.01	0.26 ± 0.01	4	149	19F	Daptomycin	
SP2020	0.96 ± 0.02	0.98 ± 0.01	4	27	T4	-	GntR family transcriptional regulator / 3 / Regulator
SP2020	0.70 ± 0.04	0.86 ± 0.01	4	27	T4	Daptomycin	
SP2036	1.00 ± 0.04	1.00 ± 0.01	3	71	19F	-	Ascorbate-specific phosphotransferase enzyme IIa / 2 / Transport
SP2036	1.17 ± 0.05	1.02 ± 0.01	3	71	19F	Daptomycin	
SP2195	1.01 ± 0.06	0.94 ± 0.02	3	52	T4	-	Transcriptional regulator CtsR / 3 / Regulator
SP2195	1.07 ± 0.02	0.85 ± 0.02	3	55	T4	Daptomycin	
SP2195	1.03 ± 0.04	0.97 ± 0.01	3	52	19F	-	
SP2195	0.87 ± 0.06	0.96 ± 0.02	3	55	19F	Daptomycin	
SP2221	0.74 ± 0.02	n.d. ^h^	4	n.i. ^i^	T4	-	Cobalt transporter ATP-binding subunit / 2 / Transport
SP2221	0.74 ± 0.03	n.d. ^h^	4	n.i. ^i^	T4	Daptomycin	
SP2221	0.82 ± 0.05	0.83 ± 0.08	3	10	19F	-	
SP2221	0.72 ± 0.04	0.75 ± 0.10	3	7	19F	Daptomycin	

^**a**^ Knocked out gene.

^**b**^ Fitness obtained by growth curves and/or competition experiments.

^**c**^ Fitness obtained by Tn-Seq.

^**d**^ Number of independently performed competition experiments or growth curves.

^**e**^ Number of transposon insertions Tn-Seq fitness is based on.

^**f**^ Background strain in which gene was knocked out.

^**g**^ Not determined.

^**h**^ Not determined due to too little number of insertions.

^**i**^ No or not enough insertions.

### Daptomycin induces potassium loss in T4, which is mostly counteracted by a single Trk-K^+^-uptake system

**Fig 5:**
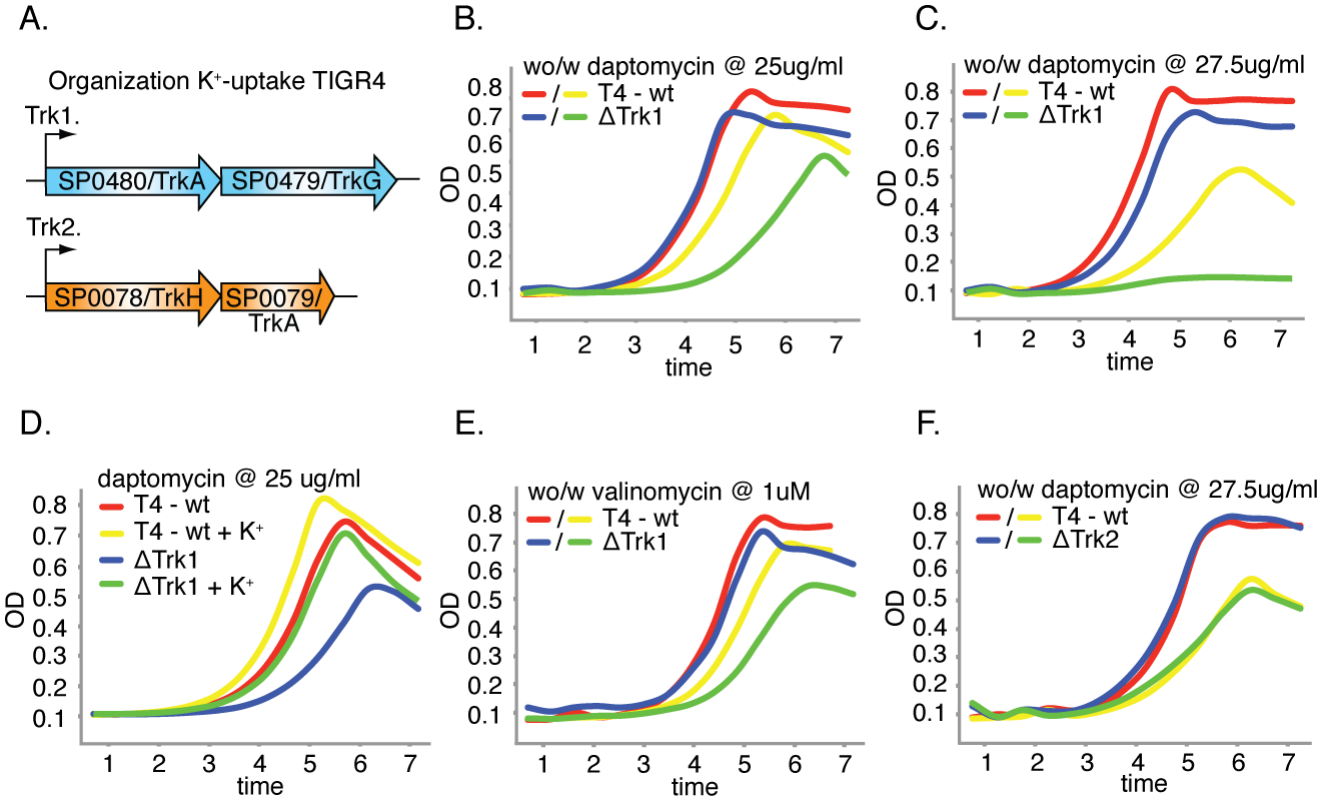
T4-K^+^-uptake mutants are sensitive to daptomycin and valinomycin. A) T4 has two Trk-K^+^-uptake-systems; system-1, encoded by genes SP0480 and SP0479, and system-2 encoded by genes SP0078 and SP0079. B) Trk1 is important for growth in the presence of daptomycin. C) This importance increases with an increasing amount of daptomycin. D) The increased sensitivity towards daptomycin can be relieved by adding 10% additional K^+^ (to a final concentration of 23mM). E) Trk1 is also important to sustain exposure to valinomycin. F) Trk2 is not important for growth in the presence of daptomycin. Red and blue lines depict the wild type and mutant strain respectively without daptomycin, while yellow and green lines depict the wild type and mutant strain respectively with daptomycin.

To develop a better understanding of how the underlying networks differ between strains we first set out to determine the role of a Trk-K^+^-uptake system which the Tn-Seq data indicates is important in the presence of daptomycin in T4 (*W*_*Trk1*_ = 0.82), while it is dispensable in 19F (*W*_*Trk1*_ = 0.98). Ion homeostasis is an essential part of life and transport systems are mandatory for ion uptake and extrusion. Although in general only traces of potassium are available in the environment it is generally the most abundant cation in bacteria and plays an essential role in for instance the maintenance of internal pH, in membrane potential adjustment, it acts as second messenger for stress signaling and it is a regulatory element for transcription control [79] [84]. Tn-Seq data for T4 indicates that 8 transporters become important in the presence of daptomycin, of which two encode a single Trk-K^+^-uptake system (Trk1: SP0479-SP0480; Fig 5A) suggesting that T4 suffers K^+^-loss upon exposure to daptomycin and that this two-gene system is important in countering that loss. The importance of Trk1 in the presence of daptomycin was validated (Fig 5B, Table 1) and is concentration dependent indicated by a further drop in fitness upon an increase in daptomycin (Fig 5C). By adding additional K^+^ both the wt and the Trk1 mutant can be (partially) compensated, which shows that the system becomes less important when K^+^ is more abundant (Fig 5D). Moreover, the mutant is also sensitive to valinomycin (an ionophore that releases K^+^) confirming that the importance of Trk1 is indeed due to daptomycin induced K^+^-loss (Fig 5E). Interestingly, *S*. *pneumoniae* T4 has an additional Trk-uptake system (Trk2: SP0078-SP0079; Fig 5A) that was not indicated by Tn-Seq (there was no loss of fitness), which we indeed verified (Fig 5F, Table 1).

**Fig 6:**
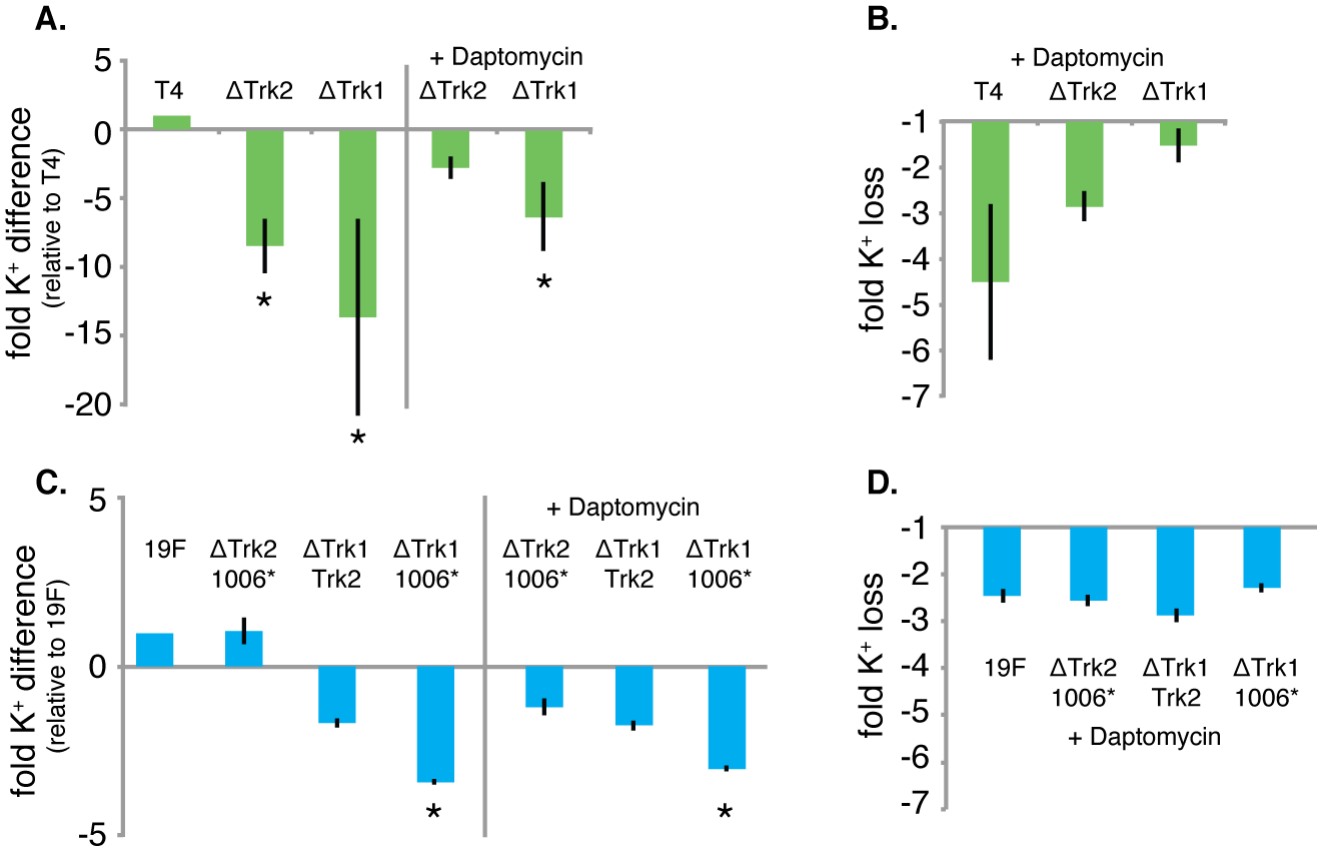
Intracellular K^+^-concentration is controlled in a strain dependent manner. A) Intracellular K^+^-concentrations and daptomycin induced K^+^-loss were measured in T4-wt and 19F-wt and deletion mutants in K^+^-uptake. A) Intracellular K^+^-concentrations for Trk1 and 2, in the absence and presence of daptomycin are shown relative to the T4-wt K^+^-concentration. In the absence of daptomycin the K^+^-concentration is significantly lower in both mutants (p < 0.002; using a student’s *t*-test with Bonferroni correction for multiple testing), while in the presence of daptomycin only the K^+^-concentration of Trk1 remains significantly lower than the wt (p < 0.001). B) The fold K^+^-loss due to daptomycin is largest for T4 followed by Trk2 and Trk1. C) Intracellular K^+^-concentrations were determined for all double mutants in 19F in the presence and absence of daptomycin. The differences in K^+^ were much smaller then in T4, and only double mutant 19F-ΔTrk2-SPT1006 has a significantly lower K^+^-concentration in the absence and presence of daptomycin relative to the wt (p < 0.001). D) The fold K^+^-loss due to daptomycin is similar for the wt and all three double mutants.

To further investigate the role of the two Trk-systems in K^+^-homeostasis as well as their importance in dealing with daptomycin stress, internal K^+^-concentrations under different conditions were determined for the wild type and the two Trk-system mutants. Although under standard growth conditions there is no clear difference in fitness between the wt, and the two Trk-system deletion mutants, internal K^+^-concentrations do vary between strains: ΔTrk2 contains on average 7-fold less K^+^, while ΔTrk1 contains on average 14-fold less K^+^ relative to the wt (Fig 6A). Interestingly, ΔTrk1 becomes very unstable under standard growth conditions and after it reaches peak OD it settles at the bottom of the culture vial, indicating that at high OD it is highly susceptible to lysis. Temporal gene expression analysis (with/without daptomycin) did not reveal any differences between the different Trk-systems. However, upon exposure to daptomycin clear differences in the loss of K^+^ were observed between all three strains: K^+^ -loss for the wt is the greatest (~4.5 fold), it is intermediate for ΔTrk2 (~2.5 fold), while for ΔTrk1 the already low K^+^ concentration drops by another 1.5-fold (Fig 6B). K^+^ -loss thus partially seems dependent on the K^+^ concentration in the cell, *i*.*e*. the higher the concentration the bigger the loss. However, even though ΔTrk1 has the smallest relative loss, its final concentration is still ~7.5 fold lower than the wild type (Fig 6A), which is enough to drastically hamper its growth (Figs 1 and 5B–5C). In contrast, ΔTrk2 only has a 2-fold lower K^+^-concentration than the wt, which is apparently not large enough to affect growth. These results show that there is a clear hierarchy in Trk-system importance; Trk1 being the most important K^+^-uptake system to control K^+^-homeostasis both during normal growth conditions as well as during exposure to daptomycin.

### K^+^-homeostasis and the response to K^+^-stress is orchestrated in a strain dependent manner

**Fig 7:**
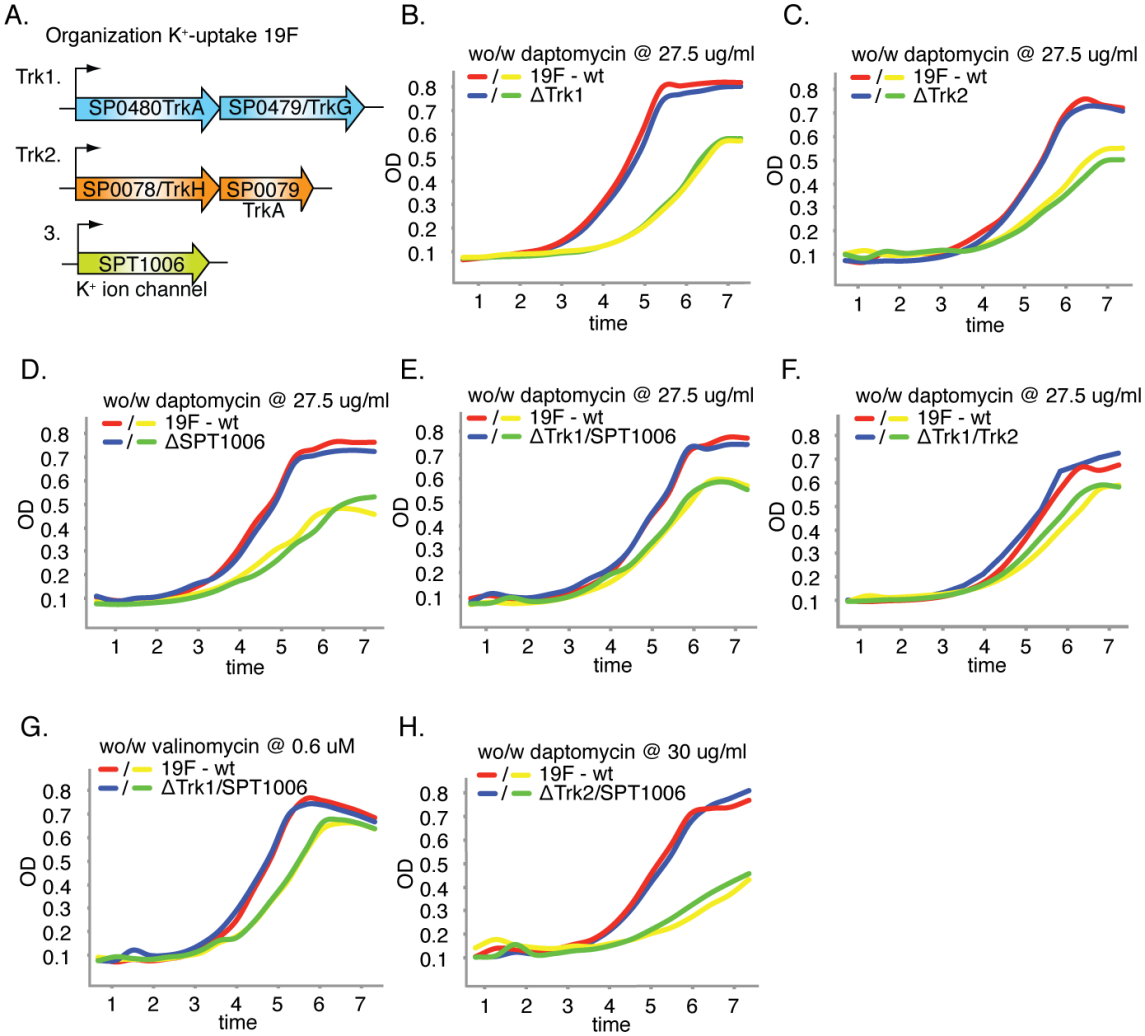
19F-K^+^-uptake mutants are not sensitive to daptomycin or valinomycin. A) 19F has three K^+^-uptake-systems; system-1 (SP0480, SP0479), system-2 (SP0078, SP0079), and system-3, which is strain-specific for 19F (SPT1006). B-D) K^+^-uptake-systems-1, 2 and 3 are not important for growth in the presence of daptomycin. E-H) Double mutants of the three systems also have no significant effect on growth in the presence of daptomycin or valinomycin (only growth in the presence of valinomycin for double mutant Trk1-SPT1006 is shown). Note that higher concentrations of daptomycin were used compared to Fig 5, to illustrate that the absence of sensitivity is independent of daptomycin concentration. H) Red and blue lines depict the wild type and mutant strain respectively without daptomycin, while yellow and green lines depict the wild type and mutant strain respectively with daptomycin.

The lack of phenotypic conservation for Trk1 with respect to daptomycin-sensitivity between T4 and 19F could be the result of a third K^+^-uptake system in 19F that creates redundancy in K^+^-control. Indeed, we found a strain-specific third system in 19F encoded by SPT1006, which is annotated as a K^+^-ion channel (Fig 7A). To determine whether there is any hierarchy among these three systems, single knockouts were created, as well as double knockouts for all three possible combinations. However, none of these six mutants made 19F more susceptible to daptomycin. Even the double knockout consisting of the strain-specific 19F K^+^-system (SPT1006) and Trk1 did not affect sensitivity of 19F to daptomycin nor valinomycin (Fig 7B–7H). Measuring internal K^+^-concentrations confirmed that daptomycin-induced release of K^+^ was similar for 19F-wt and the three single mutants (S3 Fig). The double mutants showed more variation but none of them was as dramatic as the knockout for Trk1 in T4 (Fig 6A–6D). This suggests that daptomycin-induced release of K^+^ is counteracted by all three 19F K^+^-uptake systems and that, in contrast to T4, there is no hierarchy amongst the systems. Additionally, K^+^-concentrations are approximately 5-fold higher in wt-T4 compared to wt-19F and daptomycin may have less of an impact on K^+^-loss in 19F, while instead other ions may be leaking out as suggested by the importance of the manganese transporter in 19F (Fig 2B; SP1649-1650). Importantly, these data confirm the Tn-Seq data and the lack of conservation between T4 and 19F indicating that the two strains handle K^+^-stress differently, possibly due to differences in their underlying networks.

### Genetic interaction mapping reveals a strain-dependent underlying genomic network

**Fig 8:**
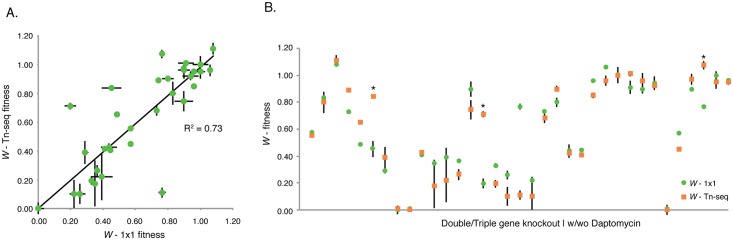
Sensitivity profile data of double knockouts correlates strongly with 1x1 competitions and growth curves. A) Correlation between Tn-Seq fitness from the Genetic Interaction Maps (GIMs) and fitness calculated from double or triple deletion mutants through growth curves or 1x1 competitions (n = 40, shown are average ± SEM). B) Three significant differences (highlighted with an *) were found between GIM-fitness data and 24 deletion mutants that were individually verified in the presence or absence of daptomycin (40 genotype-phenotype relationships; Table 2).

We have previously shown that we can (partially) reconstruct underlying networks by generating a genetic interaction map (GIM), which is accomplished by creating a transposon library in the background of a query strain (*i*.*e*. a strain that carries a knockout of the gene of interest) [46, 48]. For T4, ΔTrk1 was used as a query strain, while for 19F the double mutant ΔTrk1-ΔSPT1006 was used to compensate for the presence of the additional K^+^-ion channel. Significant response genes (*i*.*e*. interactions that deviate from the multiplicative model; see methods section for definition) were visualized in a network and grouped according to their functional category (S4A and S4B Fig; S4 and S5 Tables). There seems to be little conservation in genetic interactions: the networks are significantly different (*J* = 0.08, p<0.001), the overall distribution of functional gene categories is significantly different (*two proportion exact test*, Z = 8.11, N_1_ = 49 N_2_ = 34, p<0.01), as well as the global response (*two proportion exact test*, Z = 4.38, N_1_ = 49 N_2_ = 34 p<0.01). One of the most obvious differences between the strains is found in the second layer, which includes 18 transporters in T4, of which Trk1 and 2 are indicated as synthetically lethal, which we confirmed by our inability to construct this double mutant, five transporters have an aggravating interaction (a lower fitness than expected from the multiplicative model), while 12 interactions are alleviating (a higher fitness than expected from the multiplicative model). Moreover, a further 21 additional alleviating interactions are present between Trk1 and genes from all four layers. In contrast, there are only 5 overall conserved interactions between strains, including a single alleviating transporter interaction. Moreover, of the seven aggravating transporter interactions in 19F, six are annotated as involved in metal-ion transport, indicating that the manner in which 19F deals with daptomycin-induced loss of membrane-potential may be decentralized and spread across multiple different transporters. To confirm the genetic interactions and lack of conservation between strains we validated 21 genotype-phenotype relationships of which one (ΔTrk1-ΔSP2195) had a significantly higher fitness than expected (Fig 8A and 8B; Table 2). The GIMs are thus robust, and give hints as to the type of relationships between interacting genes. For the metal-ion transporters in either map it seems relatively easy to explain why they interact with the query genes: the removal of the K^+^-homeostasis system(s) makes both strains sensitive to a further disturbance in the bacterium’s ion-mediated potential, and thus a loss of any transporter that is involved in retaining what is left of that potential will have a negative effect on fitness. In contrast, the alleviating interactions in T4 show that the removal of these genes has a positive effect on fitness. This effect could also be accomplished by transcriptionally repressing these genes, and thus this suggests that these genes are dysregulated in a T4-ΔTrk1 background.

**Table 2:** Fitness verification of double and triple deletion mutants, with and without daptomycin.

Gene^a^	W—1x1 ± s.e.^b^	W—Tn-seq ± s.e.^c^	1x1 n^d^	Tn-seq n^e^	Strain background^f^	Condition	Annotation / Layer / Role
ΔSP0019	0.57 ± 0.02	0.56 ± 0.08	3	9	T4-Δ*clpP*	-	Adenylosuccinate synthetase / 4 / Nucleotide metabolism
ΔSP0019	0.83 ± 0.01	0.8 ± 0.04	3	11	T4-Δ*clpP*	Daptomycin	
ΔSP0019	1.08 ± 0.01	1.11 ± 0.01	3	1	19F-ΔTrk1-SPT1006	-	
ΔSP0019	0.74 ± 0.01	0.89 ± 0.01	3	1	19F-Δ Trk1-SPT1006	Daptomycin	
ΔSP0047	0.48 ± 0.06	0.65 ± 0.01	4	39	T4-Δ*clpP*	-	Phosphoribosylaminoimidazole synthetase / 4 / Nucleotide metabolism
ΔSP0047 *	0.45 ± 0.02	0.84 ± 0.08	4	39	T4-Δ*clpP*	Daptomycin	
ΔSP0047	0.29 ± 0.03	0.39 ± 0.03	4	15	19F-Δ*clpP*	Daptomycin	
ΔSP0078	0.00	0.00	-	n.i. ^h^	T4-ΔTrk1	-	Trk potassium uptake Trk2 / 2 / Transport
ΔSP0078	1.06 ± 0.06	n.d. ^g^	4	n.i. ^h^	19F-ΔTrk1	-	
ΔSP0078	0.97 ± 0.04	n.d. ^g^	4	n.i. ^h^	19F-ΔTrk1	Daptomycin	
ΔSP0078	0.00	0.00	-	n.i. ^h^	19F-ΔTrk1-SPT1006	-	
ΔSP0202	0.41 ± 0.02	0.42 ± 0.16	4	3	T4-Δ*clpP*	-	Anaerobic ribonucleoside triphosphate reductase / 4 / Nucleotide metabolism
ΔSP0202	0.35 ± 0.07	0.17 ± 0.16	4	3	T4-Δ*clpP*	Daptomycin	
ΔSP0202	0.39 ± 0.02	0.22 ± 0.04	4	25	19F-Δ*clpP*	Daptomycin	
ΔSP0205	0.52 ± 0.11	n.d. ^g^	4	n.i. ^h^	T4-Δ*clpP*	-	Anaerobic ribonucleoside-triphosphate reductase activating protein / 4 / Nucleotide metabolism
ΔSP0205	0.39 ± 0.11	n.d. ^g^	3	n.i. ^h^	T4-Δ*clpP*	Daptomycin	
ΔSP0205	0.36 ± 0.06	0.26 ± 0.07	4	6	19F-Δ*clpP*	Daptomycin	
ΔSP0798	0.90 ± 0.04	0.74 ± 0.02	4	4	T4-Δ*clpP*	-	DNA-binding response regulator CiaR / 3 / Regulator
ΔSP0798*	0.20 ± 0.02	0.71 ± 0.03	4	4	T4-Δ*clpP*	Daptomycin	
ΔSP0798	0.33 ± 0.03	0.19 ± 0.07	4	8	19F-Δ*clpP*	-	
ΔSP0798	0.26 ± 0.02	0.10 ± 0.03	4	8	19F-Δ*clpP*	Daptomycin	
ΔSP0798	0.76 ± 0.03	0.11 ± 0.10	4	5	19F-ΔTrk1-SPT1006	-	
ΔSP0798	0.22 ± 0.01	0.10 ± 0.04	4	5	19F-ΔTrk1-SPT1006	Daptomycin	
ΔSP1013	0.73 ± 0.04	0.68 ± 0.02	3	24	T4-Δ*clpP*	-	Aspartate-semialdehyde dehydrogenase
ΔSP1013	0.80 ± 0.05	0.90 ± 0.03	3	26	T4-Δ*clpP*	Daptomycin	
ΔSP1013	0.44 ± 0.01	0.42 ± 0.01	3	10	19F-Δ*clpP*	-	
ΔSP1013	0.44 ± 0.01	0.41 ± 0.02	3	10	19F-Δ*clpP*	Daptomycin	
ΔSP1013	0.96v± 0.01	0.85 ± 0.04	3	9	19F-ΔTrk1-SPT1006	-	
ΔSP1013	1.06 ± 0.05	0.96 ± 0.06	3	9	19F-ΔTrk1-SPT1006	Daptomycin	
ΔSP1398	1.00 ± 0.05	1.00 ± 0.02	3	16	T4-ΔTrk1	-	Phosphate ABC transporter permease / 2 / Transport
ΔSP1398	0.91 ± 0.04	1.01 ± 0.06	3	16	T4-ΔTrk1	Daptomycin	
ΔSP1398	0.9 ± 0.05	0.96 ± 0.03	3	10	19F-ΔTrk1-SPT1006	-	
ΔSP1398	0.94 ± 0.04	0.92 ± 0.04	3	10	19F-ΔTrk1-SPT1006	Daptomycin	
ΔSP1466	0	0	3	31	T4-ΔTrk1	Daptomycin	Hemolysin
ΔSP1466	0.57 ± 0.01	0.45 ± 0.04	3	17	19F-ΔTrk1-SPT1006	Daptomycin	
ΔSP2195	0.9 ± 0.01	0.97 ± 0.03	3	21	T4-ΔTrk1	-	Transcriptional regulator CtsR / 3 / Regulator
ΔSP2195*	0.76 ± 0.03	1.07 ± 0.05	3	26	T4-ΔTrk1	Daptomycin	
ΔSP2195	1 ± 0.02	0.95 ± 0.02	3	7	19F-ΔTrk1-SPT1006	-	
ΔSP2195	0.96 ± 0.02	0.95 ± 0.04	3	7	19F-ΔTrk1-SPT1006	Daptomycin	
ΔSPT1006	0.99 ± 0.02	n.d. ^g^	6	n.i. ^h^	19F-ΔTrk1	Daptomycin	Potassium channel / 2 / Transport

^**a**^ Knocked out gene.

^**b**^ Fitness obtained by growth curves and/or competition experiments.

^**c**^ Fitness obtained by Tn-Seq.

^**d**^ Number of independently performed competition experiments or growth curves.

^**e**^ Number of transposon insertions Tn-Seq fitness is based on.

^**f**^ Background strain in which gene was knocked out.

^**g**^ Not determined due to too little number of insertions.

^**h**^ No or not enough insertions.

* Significant difference between 1x1 and Tn-Seq fitness (p-value <0.001; using a student’s *t*-test with Bonferroni correction for multiple testing).

**Fig 9:**
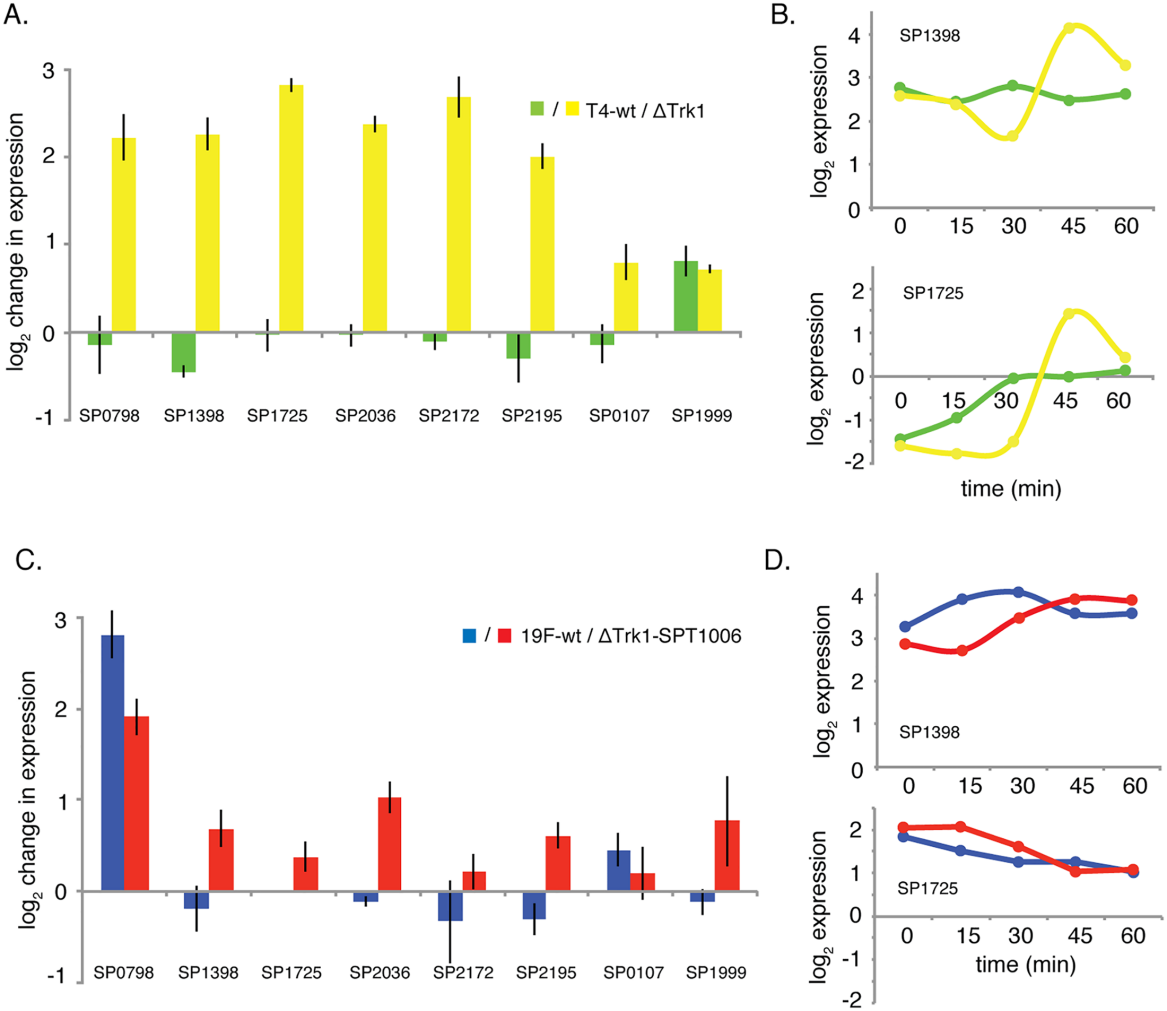
Gene-expression under K^+^-stress, changes in a strain-dependent manner. A) Expression of seven network genes and *ccpA* (SP1999), the master regulator of catabolite control [46, 100], which is not present in the network and served as a control were followed over time in T4-wt and the query strain T4-ΔTrk1. While the expression of all genes barely changes in the wt-background, six genes in the query strain background increase their expression by 4-fold between 30 and 45 minutes after the addition of daptomycin, indicating that gene-expression is dysregulated. B) Two genes are highlighted to illustrate their irregular expression in the query strain background. C) In 19F-wt and the 19F query strain expression changes are similar between 30 and 45 minutes after the addition of daptomycin, which is highlighted by temporal expression of the same two genes as in T4 (D).

To test this hypothesis we picked 7 genes from the T4-ΔTrk1 network: 4 regulators and 3 transporters. Expression of these genes was followed in wt-T4, wt-19F and the two query strains for 5 different time-points and in three independent experiments. In the query strain T4-ΔTrk1 the expression of six out of seven genes changed abruptly by approximately 4-fold between 30 and 45 minutes after addition of daptomycin (Fig 9A and 9B), while T4-wt expression did not change for any of these genes, or changes were gradual over time (Fig 9A and 9B). In 19F-wt and its query strain, expression changes for all genes were comparable (Fig 9C and 9D), and fluctuations over time, except for the response regulator *ciaR* (SP0798/TCS05) were within 2-fold. Even though the small changes in 19F could still be affecting the response, these results further show that the stress T4 and 19F experience is also processed in a different manner, seemingly with dysregulation in a select set of genes, including stress regulators such as *ciaR* and *ctsR* (SP2195), as a result.

### The role of the conserved protease ClpP is strain dependent due to differences in network connectivity

**Fig 10:**
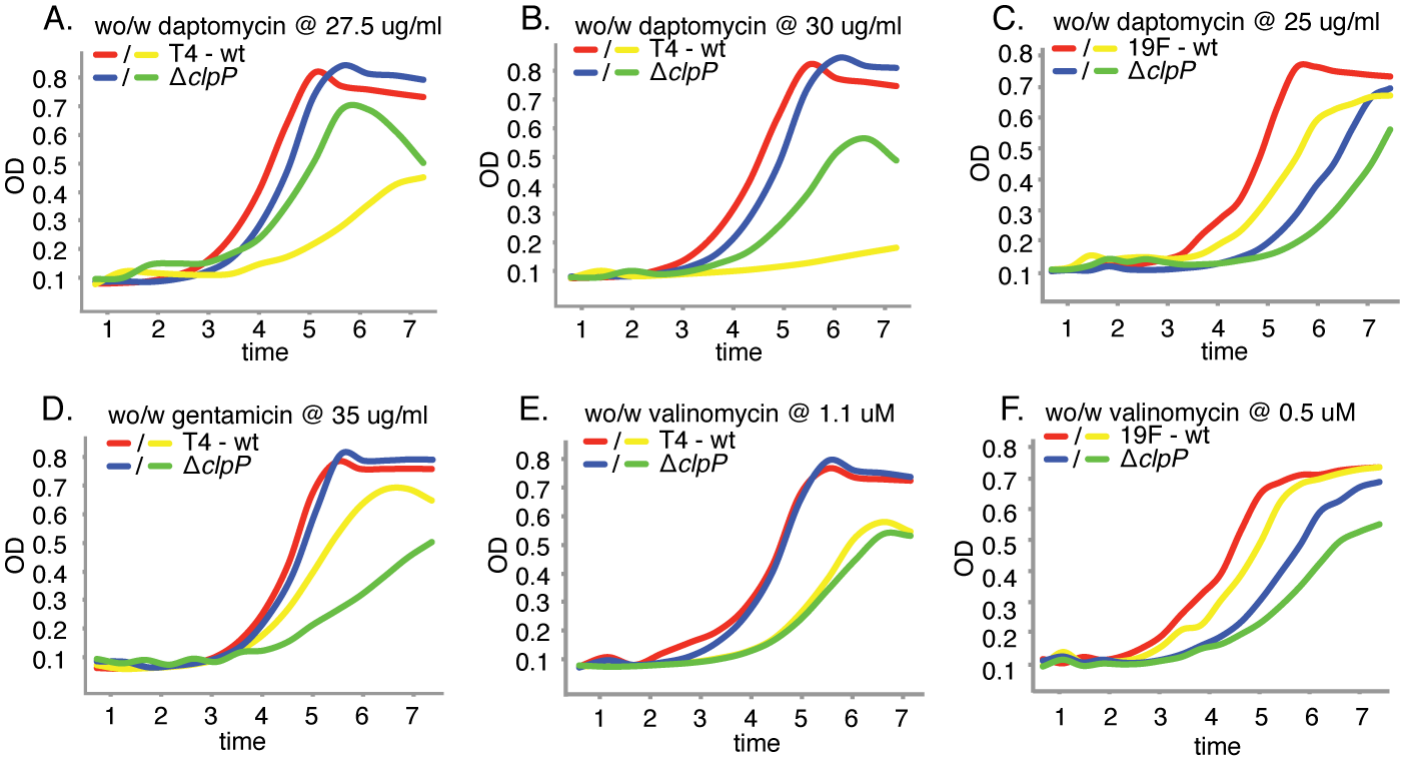
*clpP* mutants have a strain dependent effect on growth in the presence and absence of antibiotics. A) A *clpP* deletion mutant in a T4 background increases fitness in the presence of daptomycin. B) Fitness of the *clpP* mutant further increases with a higher daptomycin concentration. C) In contrast, a *clpP* deletion mutant in a 19F background decreases fitness both in the absence and presence of daptomycin. D) In the presence of gentamicin fitness is reversed, and Δ*clpP* is less fit then T4-wt. E) The phenotype seems independent of K^+^-loss since there is no change in fitness in the presence of valinomycin. F) Also this phenotype is independent of K^+^-loss since there is no change in fitness in the presence of valinomycin. Red and blue lines depict the wild type and mutant strain respectively without daptomycin, while yellow and green lines depict the wild type and mutant strain respectively with daptomycin.

One could argue that the lack of conservation in the GIMs could be unique for the K^+^-transporters, which have a relatively straightforward function and may not be that deeply integrated into the organismal network. Therefore, we set out to construct GIMs for ClpP, a protease that plays a crucial role in the regulation of various cellular responses by controlling proteolysis. For instance ClpP has been shown to repress competence in *B*. *subtilis* and activate stress proteins by targeted degradation of the repressor CtsR, additionally it has been associated with cell division, sporulation and cell wall biosynthesis [72, 85]. Thus, by definition, this conserved protease is, in its role as a protein turnover gene, deeply integrated into the organismal network. Tn-Seq analysis indicates, and individual growth curves confirm, that the role of ClpP in basic growth as well as its sensitivity to daptomycin is strain dependent: Δ*clpP* (SP0746) in T4 only slightly affects growth and, surprisingly, decreases sensitivity to daptomycin (Fig 10A and 10B; Table 1), while in 19F Δ*clpP* substantially lowers the growth rate (Fig 10C; Table 1). The T4-specifc decrease in daptomycin-sensitivity seems specific for this antibiotic, since it has the opposite effect on gentamicin sensitivity, a protein synthesis inhibitor (Fig 10D). Importantly, sensitivity to valinomycin is not affected in Δ*clpP* (Fig 10E and 10F), and thus the change in daptomycin-sensitivity does not seem to be related to intracellular K^+^-concentrations, which further confirm that the effects of daptomycin reach beyond K^+^.

**Fig 11:**
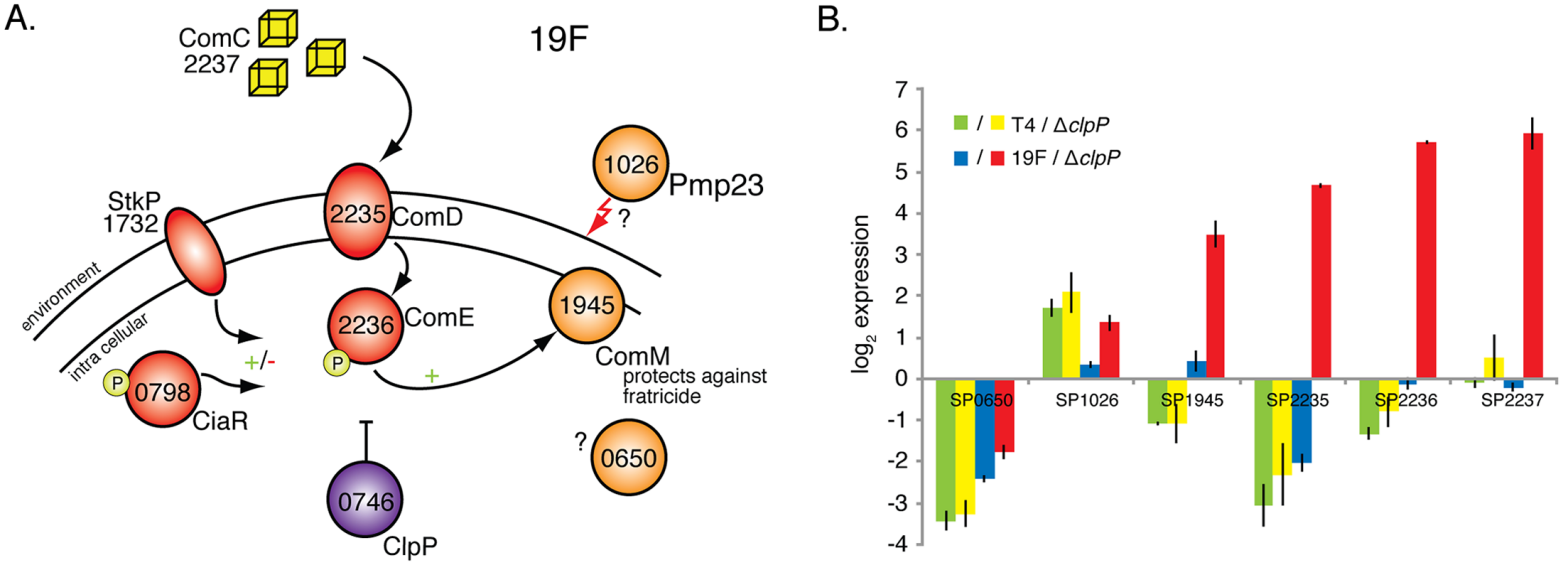
ClpP down regulates gene expression in a strain dependent manner. A) The 19F-ClpP GIM shows that ClpP interacts with a set of genes of which several play a role during competence: 1) two component system-12 (TCS12), consisting of the histidine kinase *comD* and response regulator *comE* (S5B Fig) that can be triggered by competence stimulating protein (comC/SP2237); 2) The regulators *stkP* (SP1732) and the response regulator *ciaR* (SP0798), which can regulate competence in a negative as well as positive manner [88]; and 3) *comM* (SP1945), a membrane protein that protects against lysins and fratricide [88–90]. These interactions suggest that ClpP in a 19F background has a regulatory effect on several of these genes (see main text for more details). B) The expression of four out of six genes is highly upregulated (between 12–60 fold) in the 19F-Δ*clpP* background compared to 19F-wt, while in T4-wt and T4-Δ*clpP* expression is the same. Note that expression was measured at several points in time and always showed the same pattern.

GIMs were constructed to determine ClpP connectivity within each strain as was done for the K^+^-systems (S5A and S5B Fig; S6 and S7 Tables). 19 genotype-phenotype relationships were validated; two of those relationships (SP0798-Δ*clpP*; SP0047-Δ*clpP*) have a significantly different fitness compared to Tn-Seq data, but the phenotype is stronger than initially measured, which thus confirms the interaction (Fig 8A and 8B; Table 2). Also here, there is little conservation between strains, the GIMs are significantly different (*J* = 0.06, p<0.001), the overall distribution of functional gene categories is significantly different (*two proportion exact test*, Z = 10.49, N_1_ = 23 N_2_ = 21, p<0.01), as well as the global response (*two proportion exact test*, Z = 5.06, N_1_ = 23 N_2_ = 21, p<0.05). For instance in T4, ClpP interacts in an aggravating manner with genes that are located adjacent to ClpP on the genome as well as a large number of nuclear metabolism genes. This latter relationship indicates that ClpP has control over nucleotide metabolism in T4, which has indeed been suggested for *S*. *pneumoniae* [86] as well as *B*. *subtilis* [87], but here that relationship seems to become important during daptomycin stress. In 19F, we confirmed that this strong relationship is not present (Table 2), instead, the clearest pattern in 19F emerges from ClpP interacting with a set of genes of which several play a role during competence including two component system-12 (TCS12), the regulators *stkP* (SP1732) and *ciaR* (SP0798), and *comM* (SP1945), a membrane protein that protects against lysins and fratricide [88–90] (Fig 11A, S5B Fig). The importance of TCS12 suggests that ClpP has a repressive regulatory effect on this system; *i*.*e*. since the *comD/E* system that makes up TCS12 becomes important in the absence of ClpP this suggests that it is activated (Fig 11A). To verify this, we determined expression of the two TCS12 genes, *comC* (which is in an operon with TCS12) and *comM* which is located ~300 genes downstream of TCS12. As predicted *comD*, *E* and *C*, were highly upregulated (between 20–60 fold) in 19F in the absence of ClpP (Fig 11B) while *comM* was upregulated approximately 12-fold. Because of the importance and upregulation of the ‘anti-lysis’ gene *comM*, we expected that Pmp23 (SP1026), a membrane protein that is associated with lysis through its possible role in peptidoglycan turnover [91–93], and SP0650, a membrane protein with possible hydrolase activity (which are both present in the network; S5B Fig), would also be upregulated, and that ComM was possibly protecting against their actions. However, this hypothesis had to be rejected since the expression of *pmp23* and SP0650 hardly changed in the absence of ClpP, which does not exclude that ClpP still has control over these genes through its protease activity. In contrast, relative expression of all 6 genes was unchanged in T4-wt and T4-Δ*clpP* (Fig 11B), confirming that under the tested conditions there are no relevant interactions between these genes in the T4 background. These data thus show that even for a highly conserved gene, genetic interactions are not necessarily conserved, which can lead to responses that are largely strain dependent.

### Towards a better understanding of antibiotic-resistance

It has become clear that the interaction between an antibiotic and bacterial cell is a complex, multi-factorial process that resonates through the organism requiring the involvement of a diverse set of fundamental processes to overcome the antibiotic-induced stress [5–8]. The selective pressures invoked by an antibiotic are thus not only felt by the direct target but are dispersed across many different layers. This distribution of stress thus expands the adaptive sequence space, which may explain why multiple genetic perturbations across different layers can combine to confer elevated levels of resistance [13–17]. Here, we develop daptomycin-sensitivity profiles showing in detail the genes that are important in coping with daptomycin-induced stress. By creating hierarchical layers, that partially represent physical barriers the antibiotic interacts with as well as fundamental processes that regulate and ensure all aspects of the bacterium’s life cycle, we identify where the antibiotic has its biggest impact. We believe that these types of analyses can be used to uncover the bacterium’s weakest-links in the presence of an antibiotic and thus identify novel targets that could work synergistically with existing drugs, while it also indicates where in the genome the bacterium may adapt to decrease its sensitivity to the stress. For instance, mutations in daptomycin adapted strains of *S*. *aureus*, *B*. *subtilis* and Enterococci have been observed in ClpP and other proteases, different regulatory genes and TCSs, capsule genes, transporters, nucleotide metabolism genes, peptidoglycan genes, lipoproteins and membrane genes [56, 69, 70, 73–78, 94–98]. Although many of these mutations have not (yet) been directly linked to higher resistance, we show here that they may indeed contribute to drug-sensitivity. Importantly, it turns out that the sensitivity profiles strongly depend on the genomic background, and that even within a species responses can be strain specific. We show that it is possible to at least partially dissect the underlying network of the response through constructing a GIM. By removing the query-gene, on which these maps are based, it is as if a protective layer is removed from the organism, thereby further exposing parts that become important in the presence of the stress, and at the same time revealing the type of dependencies that exist between genes, including regulatory relationships. However, by diving deeper into the response by means of these GIMs, we uncover more complexity and even less conservation across strains. Our approach thus reveals that an important part of antibiotic-induced stress is experienced and processed by *S*. *pneumoniae* in a strain dependent manner. Take this one step further and it implies that adaptation to an antibiotic will, at least partially, be strain dependent. And thus, this could be one of the reasons why it remains so difficult to predict the emergence of antibiotic resistance. This study provides, a clear approach as well as important arguments to not only construct antibiotic-sensitivity profiles for different antibiotics but also perform this across different bacterial species and strains. Such profiles in combination with *in vitro* and *in vivo* adaptation experiments could provide an important improvement in our ability to predict where in the genome mutations may arise that decrease susceptibility to an antibiotic and put the organism on the road towards full clinical-resistance.

## Methods

### Bacterial strains and genomic analysis

Experiments were performed with *S*. *pneumoniae* strains TIGR4 (NCBI Reference Sequence: NC_003028.3) and Taiwan-19F (NC_012469.1). TIGR4 is a serotype 4 strain that was originally isolated from a patient from Norway with Invasive Pneumococcal Disease (IPD) [62, 63], while 19F is a multi-drug resistant strain isolated from a patient with IPD from Taiwan [64, 65]. All gene numbers in the tables and figures are according to the TIGR4 genome, except when it concerns a strain-specific gene, these are preceded by SP or SPT referring to a T4 or 19F gene respectively. PATRIC [99] and BLAST were used to compile S8 Table, which matches gene numbers between T4 and 19F and lists strain-specific genes for each genome. A gene is considered strain-specific if 70% of the sequence has less than 70% similarity with the other genome [42]. Single gene knockouts were constructed by replacing the coding sequence with a chloramphenicol and/or spectinomycin resistance cassette as described previously [46, 48, 100]. *S*. *pneumoniae* was grown on sheep’s blood agar plates or statically in semi-defined minimal media (SDMM) at pH 7.3, which contains 70 μg/ml calcium to ensure activity of daptomycin, 20 mM glucose and 5 μl/ml Oxyrase (Oxyrase, Inc), at 37°C in a 5% CO_2_ atmosphere [48]. Where appropriate, cultures and blood plates contained 4 μg/ml chloramphenicol (Cm) and/or 200 μg/ml Spectinomycin (Spec).

### Tn-Seq experiments and fitness (*W)* analyses

Library construction was performed as described with transposon Magellan6, which lacks transcriptional terminators, therefore allowing for read-through transcription, and it diminishes polar effects [46, 48, 101, 102]. Additionally, the mini-transposon contains stop codons in all three frames in either orientation when inserted into a coding sequence. Six independent transposon libraries were constructed in wt-T4 and wt-19F and in four query strains (T4: ΔTrk1, ΔClpP; 19F: ΔTrk1-SPT1006, ΔClpP), and selection experiments were conducted in SDMM in the presence or absence of 25 μg/ml daptomycin, which in this environment moderately slows growth for both strains by ~15% (Fig 1). Sample preparation, Illumina sequencing and fitness calculations were done as described [31, 46–49, 101–103]. In short, for each insertion, fitness *W*_*i*_, is calculated by comparing the fold expansion of the mutant relative to the rest of the population by using an equation that we specifically developed to have fitness represent the growth rate of a mutant [46, 48, 103]. All of the insertions in a specified region or gene are then used to calculate the average fitness and standard deviation of the gene knockout in question. The advantage of using this approach is that *W*_*i*_ now represents the actual growth rate per generation, which makes fitness independent of time and enables comparisons between conditions and strains. To determine whether fitness effects are significantly different between conditions or strains three requirements have to be fulfilled: 1) *W*_*i*_ is calculated from at least three data points, 2) the difference in fitness between conditions has to be larger than 10% (thus *W*_*i*_—*W*_*j*_ = < -0.10 or > 0.10), and 3) the difference in fitness has to be significantly different in a one sample *t*-test with Bonferroni correction for multiple testing [46, 48]. All significant fitness values were visualized in a network with Cytoscape [66]. Importantly, here, fitness (*W*_*i*_) represents the actual growth rate per generation, which makes fitness independent of time and enables comparisons between conditions and strains. To determine whether the observed distributions in the antibiotic sensitivity profiles that are based on the functional categories or layers are different it is enough to show that, if the classes are grouped into 2 macro classes, the resulting distributions are different. To compare two 2-class distributions we use a *two proportion exact test* and we reject the equality hypothesis at a p-value ≤ 0.05. We build these two macro classes such that the differences are as large as possible. Hence, when a test cannot distinguish between these two reduced distributions it indicates that the original, non-reduced, distributions are also similar.

### Genetic interaction mapping

Genetic interactions are defined as a deviation from the multiplicative model, which states that if a strain deleted for gene i has a fitness *W*_*i*_ and a strain deleted for gene j has a fitness *W*_*j*_, then the double mutant strain *W*_*i*j_ is expected to have the fitness *W*_*i*_ x *W*_*j*_ [46, 48]. Genetic interactions were determined for the four query strains and has generally more experimental noise [46, 48], therefore to minimize false positives, we set more stringent cut offs: 1) fitness needs to be composed of at least five data points; 2) expected and observed fitness have to deviate by at least 17.5%, and 3) significant interactions have to pass a student’s *t*-test with Bonferroni correction for multiple testing.

### Potassium release experiments

An exponentially growing culture was washed and resuspended in TA buffer to an OD600 of ~0.3, and a small amount of culture was plated on blood agar for enumeration. The external background potassium concentration was measured every 3 seconds for one minute at room temperature using the MI-442 K^+^-ion microelectrode and the MI-409 dip-type reference microelectrode (Microelectrodes, Inc., Bedford, NH). Note that: 1) longer measurements proved unnecessary as readings stabilized after several seconds, and 2) for every set of measurements the electrode was first calibrated with known concentrations of KCl to ensure a linear regression (V_meas_ = mlog10[K^+^] + z), where V_meas_ is the average mV of 20 data points measured over one minute. In samples for which the effect of daptomycin on K^+^-loss was determined cells were exposed to daptomycin for 20 minutes after which they were washed and resuspended in TA-buffer. For each sample, the internal K^+^-concentration was determined in a second measurement after lysing all cells through boiling. External and internal K^+^ concentrations were calculated by converting Log10 [K^+^] into molar concentrations of K^+^ as described previously [104].

### Competition assays and single strain growth

For 1x1 competitions two strains were mixed in a 1:1 ratio and grown for approximately 8 generations to late exponential growth phase. Fitness, representing the growth rate, was calculated through the same approach as Tn-Seq data above by determining the expansion of the competition over the experiment and by determining the ratios of the competing strains at the start and at the end of the competition by plating appropriate dilutions on blood agar plates with selective antibiotics [46, 48]. Mutants were always competed against their background strain: strains with a single gene knockout were competed against the wild type strain, while double mutants were competed against the query strain. Each competition was performed no less than four times, while single strain growth was performed no less than three times in 96-well plates by taking OD_600_ measurements every half hour using a Tecan Infiniti Pro plate reader (Tecan). Additionally, competition assays and single strain growth were performed in the absence and presence of varying concentrations of daptomycin to determine whether growth rates changed with increasing concentration according to expectations (Fig 1), which was always the case. Lastly, figures throughout the manuscript depict a typical growth curve for the specific condition or mutant, while Tables 1 and 2 list growth rates calculated over all experiments.

### Expression analysis

RNA was isolated from cultures at different times using the Qiagen RNAeasy kit (Qiagen). RNA was treated with the TURBO-DNAfree kit (Ambion), after which cDNA was generated from 1 μg RNA with iScript complete kit (BioRad) and random hexamers. Quantitative PCR was performed using a BioRad MyiQ. Each sample was measured in both technical and biological triplicates, and samples were normalized against the 50S ribosomal gene SP2204.

### Data access

Tn-Seq sequencing data is deposited at the Sequence Read Archive under BioProject PRJNA318012.

## Supporting Information

S1 Fig Phylogenetic *Streptococcus pneumoniae* tree. Phylogenetic tree generated for 44 *S*. *pneumoniae* genomes, with *Streptococcus mitis* as an outgroup, which is identical to a tree published by Donati and colleagues [42]. T4 and 19F are highlighted in red. While T4 and 19F seem to be placed relatively distant from each other, the tree is based on SNPs of the core genome and only contains pneumococcal genomes that are fully closed by sequencing. With respect to genomic content two pneumococcal strains may differ by 15%, and thus the amount of variation in the presence and absence of genes between these two strains (1711 shared genes; T4 has 324 genes that are absent in 19F, and 19F has 204 genes that are absent in T4) is representative of what can be found between different strains within the species [42, 43].
(PDF)

S2 Fig Strain-specific genome composition. All genes for each strain were split into 17 functional categories. For each strain the outer circle represents the conserved genes, while the inner circle represents the distribution of unique genes. Even though there is substantial variation between strains, the overall distributions of each category, except unknown genes, are similar (N.B. For absolute numbers and percentages for each category see S1 Table).
(PDF)

S3 Fig Potassium-loss is similar for single gene knockouts of three K^+^-uptake systems in 19F. The fold K^+^-loss due to daptomycin is similar for 19F-wt and all three single knockout mutants.
(PDF)

S4 Fig Strain-specific genetic interaction maps for K^+^-control. The GIMs were constructed in a T4 background (A) in which Trk1 (SP0479) was deleted, or in a 19F background (B) in which Trk1 and the additional K^+^-uptake system-3 (SPT1006) were deleted. All edges thus now represent genetic interactions between the query gene and the rest of the genome. Color-coding and highlighting is the same as in Fig 2, except that a red edge indicates a synthetic lethal interaction, a black edge indicates an aggravating interaction, while a green edge indicates an alleviating interaction.
(PDF)

S5 Fig ClpP genetic interaction maps are strain-specific. The GIMs were constructed in a T4 (A) and 19F (B) background in which ClpP was deleted. All edges thus represent genetic interactions between the query gene and the rest of the genome. Color-coding and highlighting is the same as in Fig 2 and S4 Fig.
(PDF)

S1 Table Genomic content distribution. (DOCX)

S2 Table Tn-Seq T4-wt Fitness data. (XLSX)

S3 Table Tn-Seq 19F-wt Fitness data. (XLSX)

S4 Table Tn-Seq T4-ΔTrk1 Fitness data. (XLSX)

S5 Table Tn-Seq 19F-ΔTrk1-SPT1006 Fitness data. (XLSX)

S6 Table Tn-Seq T4-ΔclpP Fitness data. (XLSX)

S7 Table Tn-Seq 19F-ΔclpP Fitness data. (XLSX)

S8 Table T4-19F Correspondence table. (XLSX)
