# Supplementary figures and images for: Strain Dependent Genetic Networks for Antibiotic-Sensitivity in a Bacterial Pathogen with a Large Pan-Genome
Source: PLoS Pathog. 2016 Sep 8;12(9):e1005869. doi: 10.1371/journal.ppat.1005869 (PMC5015961; doi:10.1371/journal.ppat.1005869)

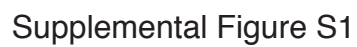

Supplemental Figure S1

Supplement: S1 Fig — Phylogenetic tree generated for 44 S. pneumoniae genomes, with Streptococcus mitis as an outgroup, which is identical to a tree published by Donati and colleagues [42]. T4 and 19F are highlighted in red. While T4 and 19F seem to be placed relatively distant from each other, the tree is based on SNPs of the core genome and only contains pneumococcal genomes that are fully closed by sequencing. With respect to genomic content two pneumococcal strains may differ by 15%, and thus the amount of variation in the presence and absence of genes between these two strains (1711 shared genes; T4 has 324 genes that are absent in 19F, and 19F has 204 genes that are absent in T4) is representative of what can be found between different strains within the species [42, 43]. (PDF) [file ppat.1005869.s001.pdf]

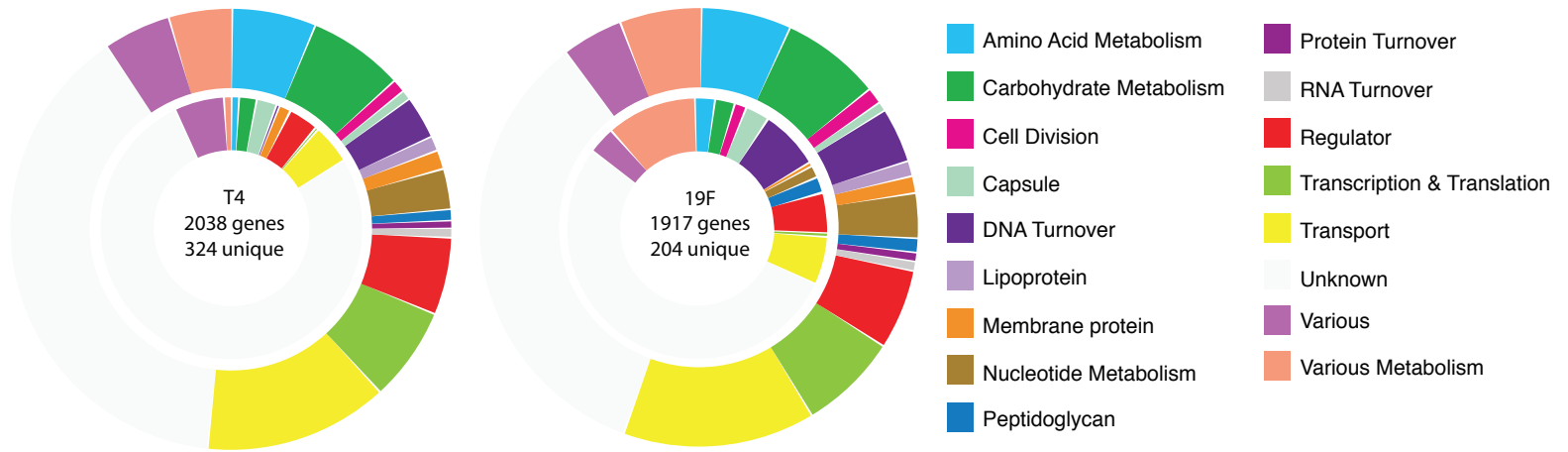

Supplemental Figure S2.

Supplement: S2 Fig — All genes for each strain were split into 17 functional categories. For each strain the outer circle represents the conserved genes, while the inner circle represents the distribution of unique genes. Even though there is substantial variation between strains, the overall distributions of each category, except unknown genes, are similar (N.B. For absolute numbers and percentages for each category see S1 Table). (PDF) [file ppat.1005869.s002.pdf]

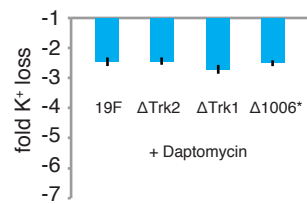

Supplementary Figure S3.

Supplement: S3 Fig — The fold K+-loss due to daptomycin is similar for 19F-wt and all three single knockout mutants. (PDF) [file ppat.1005869.s003.pdf]

A.

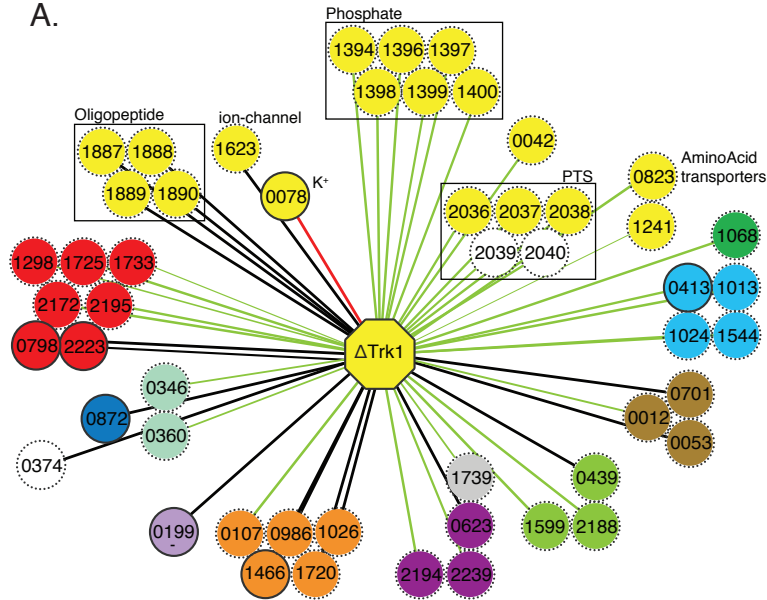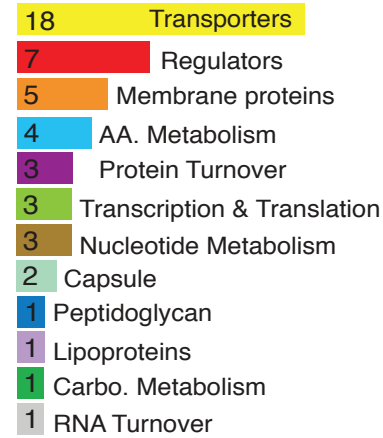

T4- $\Delta Trk1$

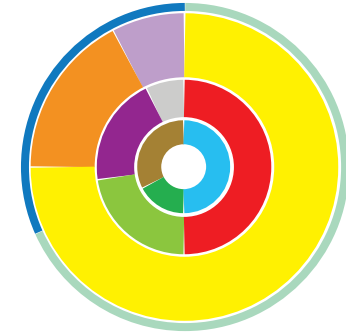

B.

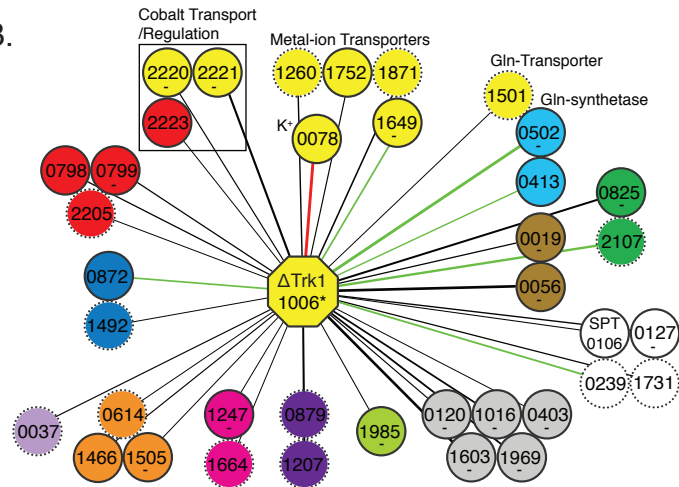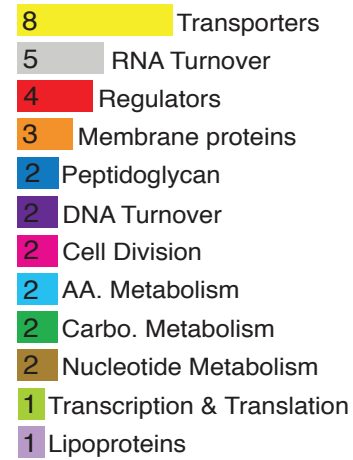

19F- $\Delta Trk1$ -SPT1006

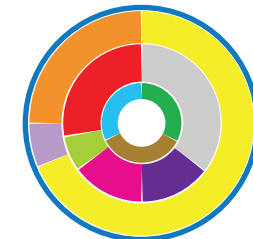

Supplemental Figure S4.

Supplement: S4 Fig — The GIMs were constructed in a T4 background (A) in which Trk1 (SP0479) was deleted, or in a 19F background (B) in which Trk1 and the additional K+-uptake system-3 (SPT1006) were deleted. All edges thus now represent genetic interactions between the query gene and the rest of the genome. Color-coding and highlighting is the same as in Fig 2, except that a red edge indicates a synthetic lethal interaction, a black edge indicates an aggravating interaction, while a green edge indicates an alleviating interaction. (PDF) [file ppat.1005869.s004.pdf]

A.

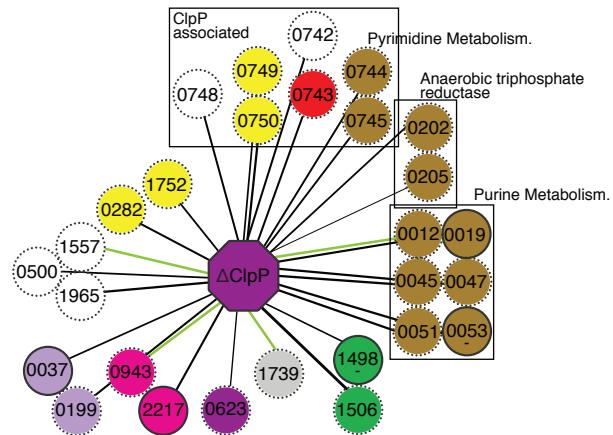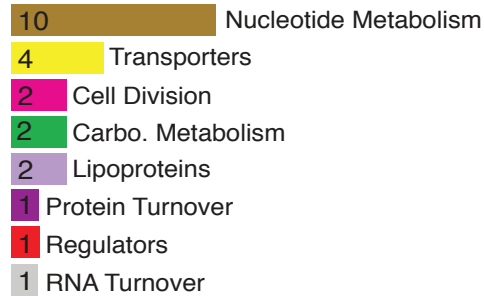

T4-ΔCIP

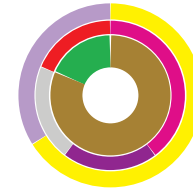

B.

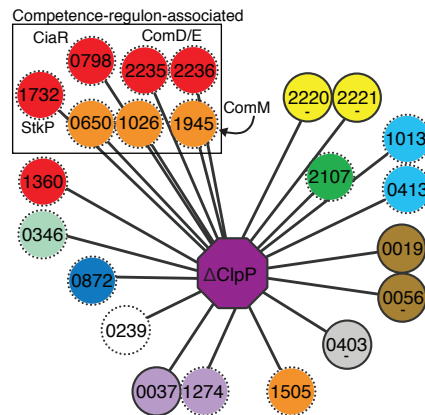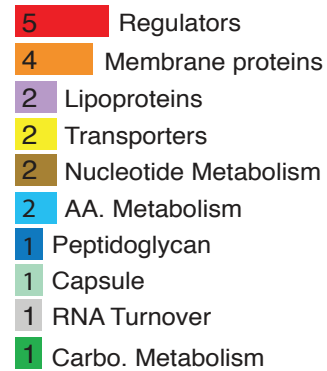

19F-ΔCIP

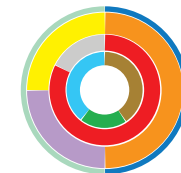

Supplemental Figure S5.

Supplement: S5 Fig — The GIMs were constructed in a T4 (A) and 19F (B) background in which ClpP was deleted. All edges thus represent genetic interactions between the query gene and the rest of the genome. Color-coding and highlighting is the same as in Fig 2 and S4 Fig. (PDF) [file ppat.1005869.s005.pdf]
