# Supplementary material for: Strain Dependent Genetic Networks for Antibiotic-Sensitivity in a Bacterial Pathogen with a Large Pan-Genome
Source: PLoS Pathog. 2016 Sep 8;12(9):e1005869. doi: 10.1371/journal.ppat.1005869 (PMC5015961; doi:10.1371/journal.ppat.1005869)
Supplement: S1 Table — (DOCX) [file ppat.1005869.s006.docx]

|  | **T4** | | **19F** | |
| --- | --- | --- | --- | --- |
| **Functional Categories** | **Total genes** ^b^ | **Strain- Specific** ^a^ | **Total genes** | **Strain- Specific** |
| Amino Acid Metabolism | 6.18% (126) | 0.93% (3) | 6.68% (128) | 2.45% (5) |
| Carbohydrate Metabolism | 7.02% (143) | 2.16% (7) | 7.36% (141) | 2.45% (5) |
| Cell Division | 0.98% (20) | 0.00% (0) | 1.20% (23) | 1.47% (3) |
| Capsule | 0.74% (15) | 2.47% (8) | 0.68% (13) | 2.94% (6) |
| DNA Turnover | 3.14% (64) | 0.31% (1) | 4.07% (78) | 7.35% (15) |
| Lipoprotein | 1.08% (22) | 0.00% (0) | 1.15% (22) | 0.00% (0) |
| Membrane Protein | 1.37% (28) | 1.54% (5) | 1.25% (24) | 0.49% (1) |
| Nucleotide Metabolism | 2.99% (61) | 0.00% (0) | 3.34% (64) | 1.47% (3) |
| Peptidoglycan | 0.83% (17) | 0.00% (0) | 1.10% (21) | 1.96% (4) |
| Protein Turnover | 0.59% (12) | 0.00% (0) | 0.63% (12) | 0.00% (0) |
| RNA Turnover | 0.69% (14) | 0.00% (0) | 0.73% (14) | 0.00% (0) |
| Regulator | 5.69% (116) | 3.70% (12) | 5.95% (114) | 4.90% (10) |
| Transcription And Translation | 6.92% (141) | 0.31% (1) | 7.36% (141) | 0.49% (1) |
| Transport | 13.59% (277) | 4.94% (16) | 14.31% (274) | 5.88% (12) |
| Unknown | 38.67% (788) | 76.54% (248) | 33.89% (649) | 53.43% (109) |
| Various | 4.91% (100) | 6.17% (20) | 4.44% (85) | 3.43% (7) |
| Various Metabolism And Biosynthesis | 4.61% (94) | 0.93% (3) | 5.95% (114) | 11.27% (23) |
| **Total number of genes** ^b^ | 100% (2038) | 15.90% (324) | 100% (1917) | 10.65%(204) |

**Supplemental Table 1. Genomic content distribution.**

^a^ Strain-Specific refers to genes that are present in one strain and absent in the other, while percentages in individual categories are based on the total number of strain-specific genes.

^b^ Percentages are based on the total number of genes in the genome.
